# Typing expertise in a large student population

**DOI:** 10.1186/s41235-022-00424-3

**Published:** 2022-08-05

**Authors:** Svetlana Pinet, Christelle Zielinski, F.-Xavier Alario, Marieke Longcamp

**Affiliations:** 1 BCBL, Basque Center on Cognition, Brain and Language, Paseo Mikeletegi 69, 2º, 20009 Donostia-San Sebastián, Spain; 2 ILCB - Institute of Language, Communication and the Brain, Aix Marseille Univ, Aix-en-Provence, France; 3 CNRS, LPC, Aix Marseille Univ, Marseille, France; 4 CNRS, LNC, Aix Marseille Univ, Marseille, France; 5 Ikerbasque, Basque Foundation for Science, Bilbao, Spain

## Abstract

Typing has become a pervasive mode of language production worldwide, with keyboards fully integrated in a large part of many daily activities. The bulk of the literature on typing expertise concerns highly trained professional touch-typists, but contemporary typing skills mostly result from unconstrained sustained practice. We measured the typing performance of a large cohort of 1301 university students through an online platform and followed a preregistered plan to analyse performance distributions, practice factors, and cognitive variables. The results suggest that the standard model with a sharp distinction between novice and expert typists may be inaccurate to account for the performance of the current generation of young typists. More generally, this study shows how the mere frequent use of a new tool can lead to the incidental development of high expertise.

**Supplementary Information:**

The online version contains supplementary material available at 10.1186/s41235-022-00424-3.

## Significance statement

Typing has become a pervasive mode of language production worldwide, with keyboards fully integrated into many daily activities. Many people, including university students, spend several hours a day typing. Such intensive practice may lead to high levels of achievement, perhaps comparable with those that professional typists had before the advent of personal computers, despite the fact that contemporary typing often relies on informal learning and accommodates a greater range of typing habits. Our preregistered study aimed at characterizing the more variable expertise currently prevalent in university students, by combining two complementary approaches to the study of expertise. The first focuses on identifying the habits that are associated with proficient performance. The second focuses on identifying the underlying cognitive processes that might differ between the most and least proficient individuals. The results show that using a keyboard frequently can, by itself, lead to the development of high expertise, and that the difference between the least and the most proficient typists is more quantitative than qualitative. The available database provides a useful benchmark for future experimental research on typing.

## Introduction

The acquisition of typing expertise has seen a radical change in the last two or three decades, going from the formal systematic training of a very limited population of professionals to a variable, often disorderly and unconstrained process carried out by a wide portion of the general population. From the invention of typewriters at the end of the nineteenth century to roughly the end of the 1980s, typewriting was almost exclusively performed by trained professionals. These individuals acquired highly homogeneous skills through intense formal training, which consisted in learning strictly systematic finger-to-key mappings, dispensing from the need to look at their hands while typing, among other requirements. This population of so-called touch-typists has received considerable attention in the scientific literature (e.g. Cooper, 1983; Gentner, 1983a), with much of this research being based on chronometric measures of performance, such as number of words per minute (wpm), response times (RT, the time elapsing between a stimulus and the first keystroke), or inter-keystroke intervals (IKI, time elapsing between two keystrokes).

Since the advent of personal computers and their progressive dissemination from the 1990s onwards, an ever-increasing population has regular access to keyboards. Typing skills have become more widespread but also more variable (Feit et al., 2016) as there might be “more than one way to speed up a typist” (Behmer & Crump, 2016). For many typists, high levels of typing performance are achieved through unconstrained sustained practice—or “experience” (Grabowski, 2008). This is most probably the case in France, for example, where typing is still only alluded to in school curricula (French Ministry of National Education, 2021). More generally, the importance given to typing in academic curricula varies substantially across countries; for instance, in the UK, the USA, or Norway, typing is a central aspect of learning to read and write (Genlott & Grönlund, 2013; Trageton, 2005).

**Fig. 1:**
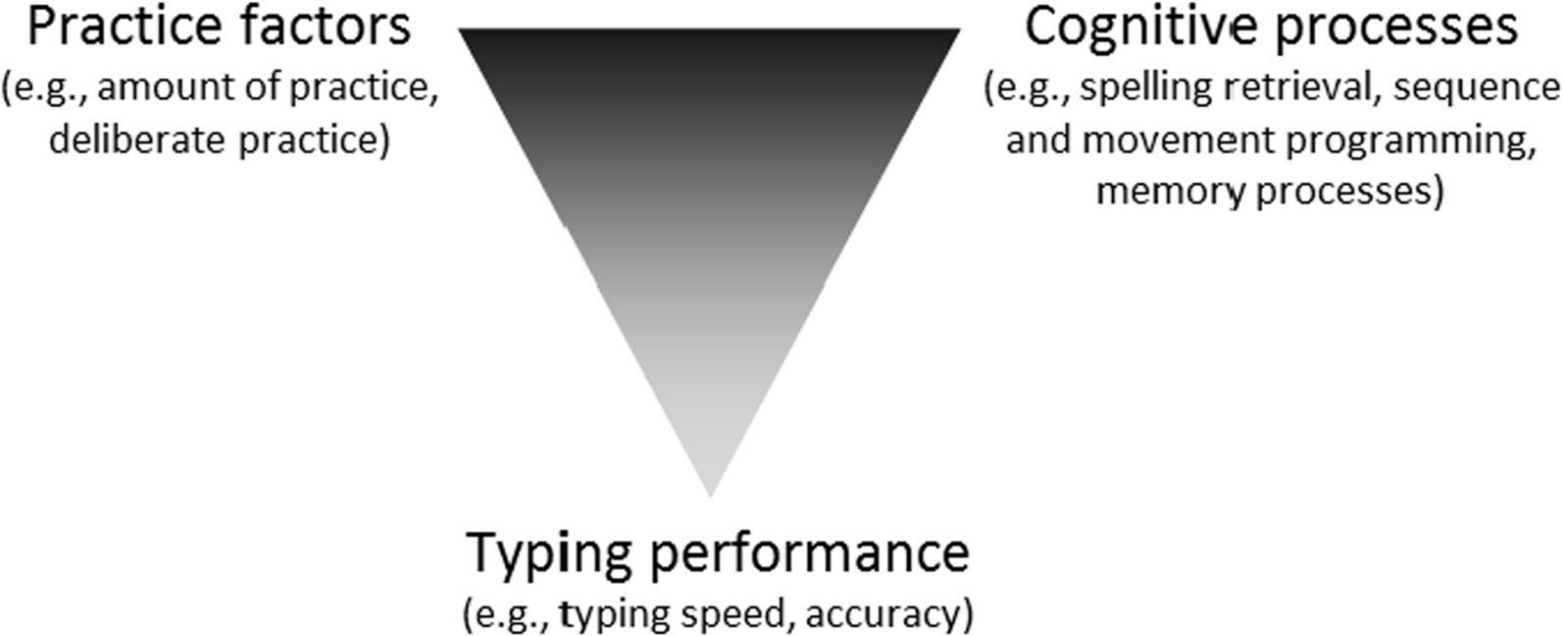
Characterizing typing performance, in terms of practice factors and cognitive processes

A thorough assessment is thus required to understand what characterizes the range of typing expertise occurring in the current population of twenty-first century typists. Two complementary perspectives can be taken on this issue (see Fig. 1). One focuses on characterizing the habits that lead to, or, minimally, are associated with, proficient typing skills. The other focuses on identifying underlying cognitive processes that might differ between the most and least proficient individuals. Our goal in the current study was to combine these two approaches, in order to determine how various practice habits and cognitive factors are related to the level of achievement in typing (Fig. 1).

As an example of the first approach, Keith and Ericsson (2007) (for a general perspective see Ericsson, 2014) explored the factors determining typing performance in a group of 60 experienced but non-professional intermediate-level typing students. The primary goal of the experiment was to assess the impact of typing habits and general abilities on typing proficiency. Participants' performance was measured in words per minute (wpm) on several tasks involving meaningful or meaningless verbal materials. This indicator of typing skills was regressed on the results of targeted interviews aimed at characterizing past and present typing habits, and of tests aimed at characterizing general cognitive, perceptual, and motor skills. In these data, the most determining factor for performance was a form of deliberate practice, namely training with the general explicit goal of typing fast. The number of keystrokes accumulated since the beginning of practice (i.e. the actual typing experience) also affected performance. In contrast, the individuals' general cognitive, perceptual, and motor skills did not show significant effects in these data (Keith & Ericsson, 2007). The authors concluded that intermediate and expert performance may be served by the same mechanisms, and that better performance is linked to the active motivation to improve.

More recently, two studies described the performance distribution and typing strategies in large samples of typists taken from the general population. The observed distribution of performance was continuous, not multimodally separated in distinct levels of expertise. A high level of performance was related to more systematic finger-to-key mappings (Feit et al., 2016) and to the use of more fingers to type (Dhakal et al., 2018). In contrast to Keith and Ericsson (2007), however, Feit et al. found no effect of formal training on performance.

**Fig. 2:**
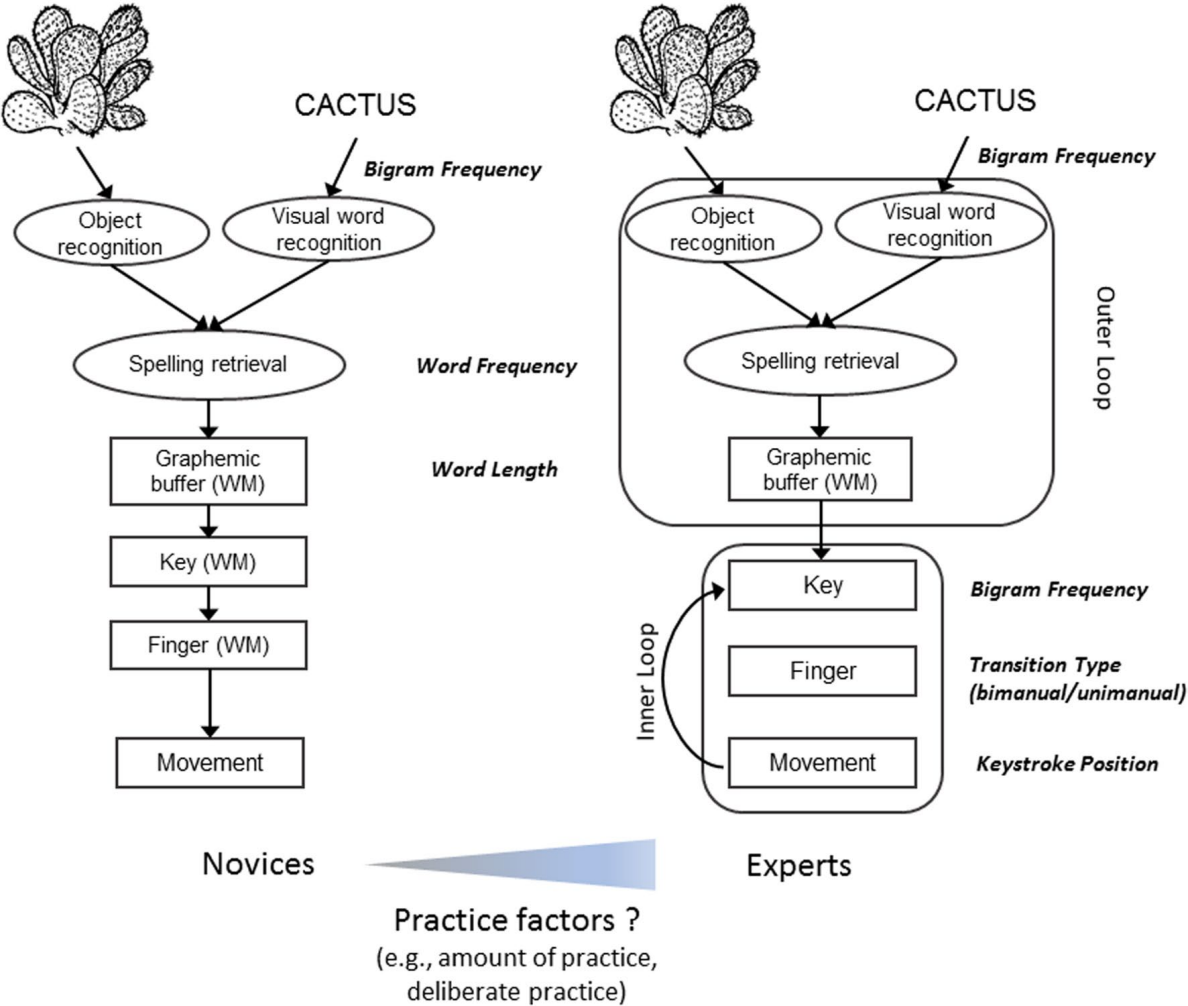
Cognitive steps of word production in novice and expert typists. Experimental variables manipulated and their hypothesized locus are depicted in bold italics. Within this framework, our main assumption is that factors of the typed material that target the motor structure of the sequences of keystrokes should impact differently the performance of the most and least proficient typists of the sample, whereas factors that affect earlier stages of language production should have a similar effect on the performance of both groups of typists. Word frequency and word length should modulate reaction times; bigram frequency, transition type, and keystroke position should modulate IKIs, and bigram frequency should modulate reaction times only in the copying task. Figure freely adapted from Alario et al., (2004), Bonin et al. (2003), and Logan and Crump (2011)

In the second approach, the focus is on the cognitive architecture of typing skills. An influential account has been put forward and refined over the years by G. Logan and his collaborators (Logan & Crump, 2011). Experts’ processing is characterized by their automation of the motor steps of keystroke sequencing and execution. This results in a hierarchical organization of the typing skill, with two independent control loops that enable (i) retrieving from memory the words to be typed (outer loop), and (ii) striking the corresponding keys in a fully automatic fashion (inner loop). Conversely, in novices, typing is thought to rely much more on the support of working memory throughout the processes from planning to striking the appropriate keys (Logan, 2018; Logan et al., 2016) (Fig. 2).

This type of model stems from early studies of typing where it was found that several features of the typed material influence typing performance. For typists who use systematic mappings between fingers and keys, successive keystrokes performed with the same hand (so-called uni-manual transitions, that is transitions between letters located on the same side of the keyboard) show longer IKIs than those performed with different hands, presumably because of parallel planning of actions in the latter case (Coover, 1923; Gentner, 1983a; Kinkead, 1975; Larochelle, 1983; Ostry, 1983; Terzuolo & Viviani, 1980). This effect is probably the most prevalent feature of typing expertise, and it is much more rarely observed in novices (Gentner, 1983a, 1983b; Larochelle, 1983). The frequency of bigrams (i.e. pairs of letters) in the written language has also been identified as an important discriminating factor, with stronger facilitating effects on expert than on novice typing performance (Behmer & Crump, 2016; Cerni et al., 2016a, 2016b; Gentner et al., 1988; Grudin & Larochelle, 1982; Ostry, 1983; Salthouse, 1986; Terzuolo & Viviani, 1980). Conversely, the length in letters of the word to be typed has a stronger positive effect on the RT in novices than in professional typists (Gentner, 1983b; Larochelle, 1983; Sternberg et al., 1978). Gentner (1983b) interpreted these observations as the result of performance shifting from being limited by cognitive constraints in novices, to being limited by motoric constraints in experts.

More recently, Behmer and Crump (2016) reported a large scale online study performed by 400 twenty-first century typists who varied naturally in skill level, with roughly half of them having followed formal training. In the data, typing skill (operationalized as the mean IKIs recorded during paragraph copying) was correlated with the sensitivity to the sequential structure of the language (operationalized as the effect of bigram or trigram frequency on IKIs). The co-variation patterns of these two variables suggested that the learning process derives from general memory processes, such as those implemented in instance theories of memory formation and retrieval (Behmer & Crump, 2016).

### The current study

Our study aimed at identifying the habits that are associated with proficient typing performance and the underlying cognitive processes that might differ between the most and least proficient individuals. The research questions, operational hypotheses, and detailed methods of this study were preregistered with the Open Science Foundation (Pinet et al., 2016). Typing expertise in a large student population. Available at osf.io/u7r36).

We assessed the typing skills of a large sample of young adults recruited among the students of our university, arguably representative of the de facto “default” population studied in many experimental psychology studies. In the studied population, typing classes are either minimal or absent from the standard curricula. To collect data from a large sample of participants, we designed an online experiment using a previously developed online platform, after having thoroughly assessed its specific reliability for measuring the timing of keystroke sequences (Pinet et al., 2017). All participants performed a sentence copying task, a picture naming task, and a single-word copying task whose order was counter-balanced across participants. After completing these tasks, participants filled out a questionnaire about their typing habits. Typing performance was quantified through the typical indexes of typing: words per minute (WPM), inter-keystroke intervals (IKIs), reaction times (RT), and accuracy rates.

We defined the two performance groups by measuring the range of typing performance in the sentence copying task (Logan & Zbrodoff, 1998). We then tested whether typing practice and habits vary across the two performance groups defined previously. We expected that high-performance typists would report spending more time typing and using more fingers than low-performance typists (Dhakal et al., 2018) and sought to determine whether high-performance typists would report deliberate practice more frequently (Keith & Ericsson, 2007) or not (Feit et al., 2016).

Finally, we tested whether the experimental manipulations known to reliably impact performance in professional typing experts would have different effects in the two subgroups of our sample. This analysis was based on data from the word copying and picture naming tasks, not previously used to define expertise groups. These tasks were used to target specific psycholinguistic processes (Baus et al., 2013; Pinet et al., 2016; Scaltritti et al., 2017), often ignored in previous studies of typewriting. In particular, the two tasks differ in their input processes (visual word and object recognition), while they share output processes (at the semantic, orthographic, and motor stages; Bonin et al., 2003). If the most proficient typists of our sample behave like expert typists, with an automatized inner loop, then their IKIs should also show effects of transition type (uni- vs. bimanual transitions between letters) and of bigram frequency. Conversely, we expect stronger effects of word length on the RTs of low- vs. high-performance typists. Given that the tasks share their output processes (see Fig. 2), these predictions are the same for the word copying and picture naming tasks. Finally, the modulation of psycholinguistic processes involved in word retrieval upstream of motor programming is expected to be similar across high and low performers. We thus expect similar effects of word frequency (Baus et al., 2013; Pinet et al., 2016) and bigram frequency (specifically in the copy-typing task; Chetail, 2015) on RTs for both proficiency groups. These cognitive processing hypotheses are summarized in Fig. 2.

## Methods

### Preregistration and ethics evaluation

Unless explicitly stated, the methods for data collection, pre-processing, and statistical analysis followed closely our preregistered protocol (https://osf.io/u7r36). The scientific and technical details of this study had been approved by the ethics committee at Aix-Marseille Universite (decision no. 2016-09-11-06).

### Participants

Participants were recruited exclusively via university listings. A recruitment email with the link to the online experiment was sent to all the students enrolled at Aix-Marseille. The email and website explicitly stated that being currently enrolled as a student at Aix-Marseille University was a requirement to participate. There were no other participant inclusion or exclusion criteria. Participants gave their informed consent online, before starting the experiment. The detection of a physical keyboard plugged to a personal computer was a necessary condition for launching the experiment. Connections via tablets or smartphones were detected and an invitation to use a personal computer was displayed instead of the experiment. Participants were informed upfront that a randomized lottery procedure will be compensating a subset of them, whereby 1 out of 50 participants will be receiving 50 EUR. To be able to claim this compensation, lottery drafted participants had to show their student ID, which certified their student status.

The preregistration included the following stop procedure for data collection: collecting data for 30 days, or until 600 participants were included, or until participation stalled (defined as fewer than 10 participants per day for 3 consecutive working days), whichever came first. Participation turned out to be much more important than we had anticipated: after a few days, we had collected data from 1504 participants, and the data collection was arbitrarily discontinued.

### Participants’ features

Among the respondents, we excluded participants that declared to be minor (28), whose self-reported native language was not French (112), who were not students (33), who did not complete the questionnaire (4), or who reported technical issues at the end of the experiment (3). Although we did not explicitly plan for it, visual inspection of performance in each of the tasks led us to exclude the data from 23 participants that did not perform the task properly in obvious ways (e.g. not providing a full answer in most trials; see Additional file 1: Appendix 2). These exclusion criteria left us with 1301 participants. In this final sample, participants who reported having followed speech therapy (203, i.e. 15%)—among which 106 participants reported a spoken disorder, 63 a written disorder, 26 both, and 8 other types of disorders—were kept in order to describe a more representative population of typists.

Out of the 1301 participants, 850 (65%) were female and 447 were male; 1062 (82%) reported being right-handed, 127 left-handed, and 110 ambidextrous. Age ranged from 18 to 69 (mean = 21.6, Q1–Q3 = 4). About half of the sample (600, 46%) reported knowing another language. In our sample, 321 (25%) reported playing or having played a musical instrument. All participants were enrolled as students when they participated, with 863 of them studying towards a Bachelor’s degree, 368 towards a Masters’ degree, and 62 towards a PhD. Their fields of study were Law or Economics (218 participants), Humanities (442), or Sciences (623).

Almost all of our sample (1188, 91%) reported typing regularly on a keyboard. Independently of the regularity of their practice, 329 reported typing on a desktop computer, 1198 on a laptop, 168 on a tablet (non-exclusive choices). Somewhat surprisingly, 531 (41%) reported using regularly keyboard configurations different from the standard French AZERTY. The typing activity reported were primarily chatting (682, 52%), note-taking (560, 43%), emailing (515, 40%), composition (429, 33%), and copying (144, 11%). Only 17 participants reported having followed some formal training to learn typing.^1^ Years of experience with typing spanned from 1 to 40 years (mean = 9.6, Q1–Q3 = 5). On their mobile device, 77 reported using phonetic spelling, 1138 reported using an AZERTY keyboard; 1242 reported using a smartphone. Regarding handwriting practices, 1135 reported writing with their right hand. On average, participants reported spending 2.0 ± 1.9 h daily typing on a computer, 1.8 ± 2.2 h on a mobile device, and 2.4 ± 2.3 h handwriting (see histograms in Additional file 1: Appendix 3).

### Materials and procedure

*Picture naming and single-word copying tasks*: 80 nouns (names of concrete and depictable objects) constituted the experimental items. Pictures were selected from various databases (Alario et al., 2004; Bonin et al., 2003; Snodgrass & Vanderwart, 1980) and other sources. Psycholinguistic and motoric variables were controlled in the following way. (1) Words were 4- to 7-letter long. (2) When typed on a French AZERTY keyboard, using standard finger-key mappings, 39 of the words started with the left hand and 41 with the right hand. (3) The proportion of hand alternations that would result from a strict observation of standard finger-key mappings was controlled. The selected items were divided into 5 groups, spanning from 0 to 100% hand alternations: 16 words with 0% transitions, 16 with 20% to 33%, 17 with 50%, 16 with 67%, and 15 words with more than 80% bimanual transitions. Note that variables (2) and (3) should only be interpreted in light of the self-reports of finger use (see discussion). (4) Mean word frequency was counter-balanced according to the laterality of the first keystroke. (5) Stimuli were selected so that the distribution of each variable was approximately uniform: word frequency, length, percentage of transitions, laterality of first keystroke, mean bigram frequency (see Additional file 1: Appendix 1). In addition, four items were selected from the same pool to be used as fillers.

Items were presented one by one on the computer screen. Each block began with four filler trials intended for task familiarization (removed from the analysis). On each trial, participants had to type the picture’s name or the word; what was typed was immediately echoed on the screen below the stimulus. Picture and word stimuli stayed on the screen until the participant finished typing and pressed the return key. The next trial started after an 800 ms inter-trial interval. Participants were given no explicit instructions on how to react in case they detected a typing error. This let them free to react as they normally would, preserving their natural behaviour in these circumstances.

*Sentence copying task:* ten sentences were selected to be presented visually (see Additional file 1: Appendix 1, Table S2). All experimental sentences were adapted from a set of university instructions explaining the procedure to set up an electronic signature in emails, a relatively elaborate yet fairly neutral content. The first sentence was treated as a task-familiarization filler item and the corresponding data were not analysed. The remaining 9 experimental sentences comprised 23 (out of the 26) letters of the French alphabet, and 167 unique bigrams out of the 676 attested bigrams in French. We ascertained that the frequency of occurrence of letters and bigrams in the text was correlated to their actual frequency in French (respectively, 0.95 and 0.67 correlations for letters and bigrams, based on the “Surface” database in New et al. (2004).

Sentences were presented one by one on the top half of the screen and remained there until participants finished typing and pressed the return key. What participants typed was immediately echoed on the screen below the target sentence. The next sentence started after an 800 ms inter-trial interval. Again, participants were given no explicit instructions regarding corrections.

*Questionnaire:* The final questionnaire comprised a sequence of 7 web-pages, with questions about general demographics and about typing and handwriting habits. The questions and possible answers are provided in Additional file 1: Appendix 1.

*Overall procedure:* Participants performed the sentence copying task, the picture naming task, and the word copying task in a random order. They completed the questionnaire about their typing habits after the three tasks. Following Pinet et al (2017), the experiment was programmed using the open-source jsPsych library version 5.0.2 (de Leeuw, 2015). The experimental scripts can be found online.

### Data analysis

The basic structure of the data file was vectors of time-stamped keystrokes (recorded online during participant performance) corresponding to the keystrokes of each word in the picture naming and word copying tasks, or to the keystrokes of each sentence in the sentence copying task. All the analysis was performed using R (version 3.3.3, R Core Team, 2013) with, most notably, the packages *lmerTest* (version 3.0–1; Kuznetsova et al., 2017), and *TraMineR* (Gabadinho et al., 2010, 2011).

**Fig. 3:**
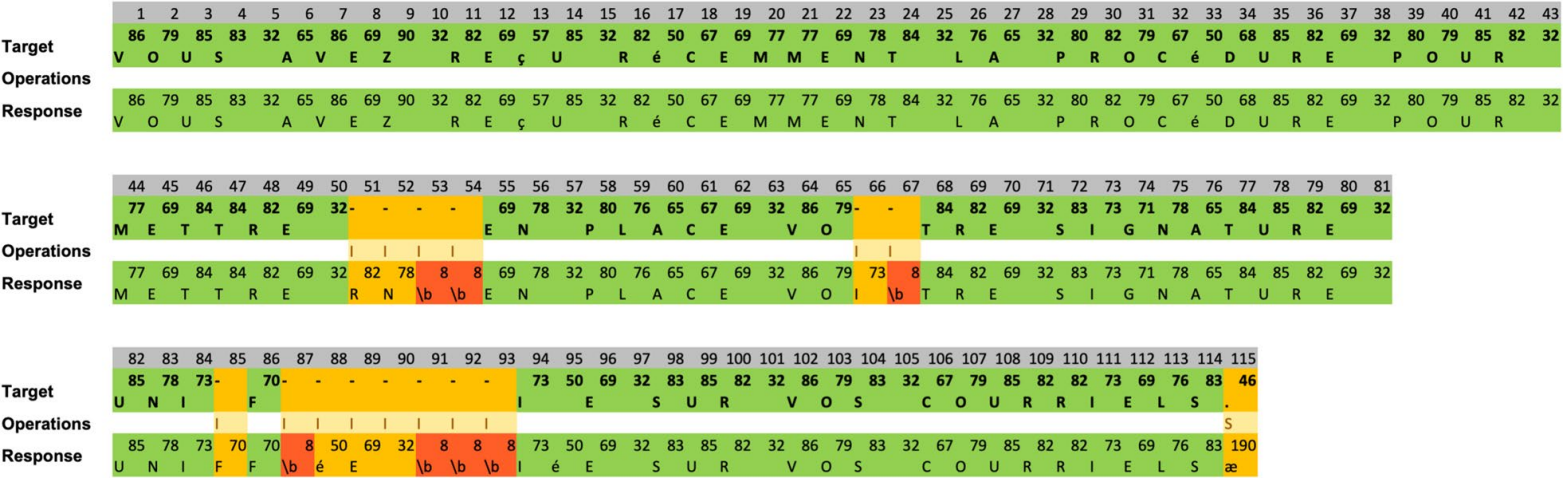
Example of quantification of accuracy through sequence comparison. Differences between the target (top row) and response sequences (bottom row) are shown as operations (I = insertion, S = substitution) in yellow, backspaces (“\b”) in red. In this example, the distance between sequences (or IDS) is 15

*Quantification of accuracy:* Accuracy was assessed offline following the preregistered procedures. For single words (word copying and picture naming tasks), the produced keystrokes were directly compared with the expected keystrokes. This made simple errors, whether corrected or uncorrected, immediately apparent as mismatches. For sentences, a more elaborate approach was adopted. This is because the sequence of keystrokes produced could deviate substantially from the expected one, despite the final result being correct or minimally deviant, due to corrections. To handle misalignment between expected and produced keystroke sequences and take into account corrections in our estimation of accuracy, we resorted to the *TraMineR* library (Gabadinho et al., 2010, 2011). For each participant and each sentence, the minimum number of insertions, deletions, substitutions (IDS) necessary to go from the target sentence to the participants’ sequence of keystrokes typed was computed using the functions “seqdef” and “seqdist”. Figure 3 shows an example sentence and the set of operations computed to obtain the IDS score.

Accuracy was computed as the difference between the total number of characters in the target sentence and the number of IDS, divided by the total number of characters in the target sentence. Responses containing less than half of the total number of characters in the target sentences were discarded as being over-erroneous (40 sentences, 0.34%). The data file is available at the same online repository.

*Quantification of timing parameters:* For single words, reaction time (RT) is defined as the time between the presentation of the stimulus and the time of the first keystroke. Inter-keystroke interval (IKI) corresponds to the time between two successive keystrokes. In the word copying and picture naming tasks, IKIs were used directly as a dependant variable. In the sentence copying task, typing speed was computed as 5-character words per minute (wpm; (Crump & Logan, 2010a), using the formula

$${\text{typing speed}} = \frac{{{\text{nb letters in text}}}}{{5*\left( {{\text{last timestamp}} - {\text{first timestamp}}} \right)}}$$

**Fig. 4:**
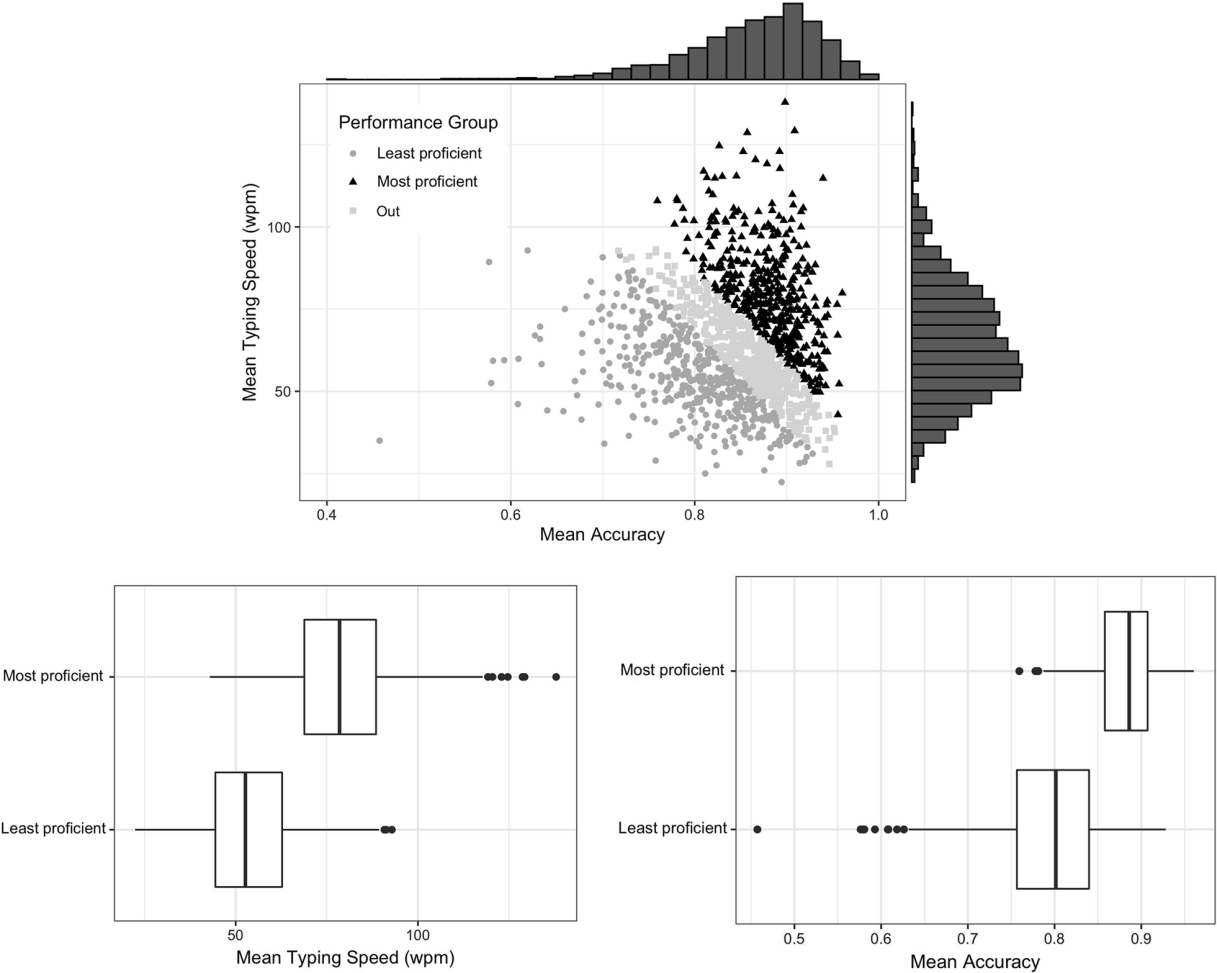
Distribution of performance in the sample. Top: summary of performance in the sentence copying task on which the definition of performance groups is based. Mean typing speed (wpm: words per minute) is plotted as a function of mean accuracy (proportion correct). Each dot is a participant. Performance groups defined following the preregistered procedure are displayed in colour/shape. Marginal distributions of typing speed and accuracy are shown on the *X* and *Y* axes. Bottom: boxplots of the typing speed and accuracy rates of the two performance groups.

*Definition of groups of participants based on their level of performance (high vs. low):* The marginal distributions of typing speeds and accuracy rates across participants did not reveal any bi- or multi-modality that would have pointed to naturally distinct populations with varying skills (Fig. 4, top panel). The same unimodality was observed in a much more diverse sample (in Dhakal et al., 2018), with data from over 200 countries, various cultural backgrounds, and a larger age range. Given the absence of naturally occurring clusters, we resorted to the second preregistered method for subdividing the population. From the bi-variate distribution of typing speed and accuracy, data below and above the median values were excluded recursively to reach ~ 33% (total exclusion, 33.3% of individuals), and the remaining two groups (each comprising ~ 33% of the population) were considered to be representative of, respectively, the least and most proficient typists, referred to as the “most” vs. “least” proficient groups. The most proficient typists had a mean typing speed of 80 wpm (IQR = 20), and an accuracy of 88% (IQR = 4.3). The least proficient typists had a mean typing speed of 54 wpm (IQR = 18) and an accuracy of 79% (IQR = 8.3). The distribution of their typing speed and accuracy rates is presented in Fig. 4, bottom panel (full table in Additional file 1: Appendix 4).

The typing speeds observed in our data were relatively high, overall. Touch-typists enrolled as participants in cognitive studies type at an average rate of 70–80 WPM (Logan, 2018; Salthouse, 1984); the overall mean typing speed was of 52 WPM in Dhakal et al. (2018). It is possible that some features of our task promoted faster typing speeds, notably the use of short individual sentences rather than full paragraphs.

The accuracy data confirm that typing behaviour is error-prone, even in individuals classified among the most proficient (Pinet & Nozari, 2021). The number of errors we observed was around 12% for the most proficient and 20% for the least proficient typists. This is considerably larger than that of typing experts and novices using typewriters for whom error rates were typically under 3.2% (Grudin, 1983; Salthouse, 1986), and even 0.3% in a study of a single typist spanning over 1.3 millions of keystrokes (Logan, 1999). Note that our measure of accuracy included corrections (see corresponding methods section), which might not be directly comparable to other studies. In modern typing, the possibility of correction through backspacing prompts the development of automatized routines for error correction (Crump & Logan, 2013).

Demographic information about the resulting groups is presented in Table 1. Linear regression analysis showed that the proficiency groups did not differ in their gender distribution, *β* = 0.193, *z* = 1.14, *p* = 0.17, and that the most proficient typists were significantly older than the least proficient, *β* = 0.937, *t* = 3.2, *p* = 0.0014.

**Table 1:** Demographic information about performance groups

	Least proficient	Most proficient
*N*	432	435
Age (years)	21 ± 4.9	22 ± 3.6
*Gender (N)*		
Female	264	284
Male	168	149

*Assessment of the link between typing habits and the performance groups:* For each numerical variable (daily time typing, years of practice, number of fingers used), we ran a linear regression model, including performance groups as predictor. A Box–Cox transform was used to determine which variables needed transformation to approach normality, resulting in the log-transformation of the variable daily time spent typing text only. For categorical variables (deliberate practice, lecture note-taking with a computer), we ran a logistic regression. Finally, for the ordinal variable (looks to keyboard), we ran an ordered logistic regression. Following a previous study showing the effect of age and gender on typing performance (Pinet et al., 2017), we included these two variables as co-variates. All models had the following structure: DV ~ performance groups + age + gender.

*Assessment of the effect of psycholinguistic and motor variables on IKIs and RTs of the copy-typing and picture naming tasks:* Individual IKIs, RTs, and accuracy rates were analysed using mixed-effect linear models, using the R package *lmerTest*. RT and accuracy were modelled using the same model structure, except that accuracy was modelled using a logistic link function. For all models, random effects of participants and items (words for RTs, bigrams for IKIs) were included. Some models would not converge when random slopes were included; for the sake of consistency, they were not included in any model. IKI were modelled with fixed effects for word frequency, length, transition type, position within word, bigram frequency, and performance groups, as well as the interaction terms for each fixed effect with performance group. The models for RT and accuracy included overall the same predictors, although taken into account over the full word (bigram frequency and transition type included as mean bigram frequency and transition ratio over the full word). Predictors were word frequency, length, transition ratio, laterality of first keystroke, mean bigram frequency, and performance groups, as well as the interaction terms of each fixed effect with the factor performance groups. Performance groups as well as other categorical variables (transition type, laterality) were coded using treatment contrast. For details on accuracy rates and their analysis, see Additional file 1: Appendix 6.

## Results

These results stem from our preregistered analysis protocol (https://osf.io/u7r36).

### Do typing habits vary according to performance groups?

**Fig. 5:**
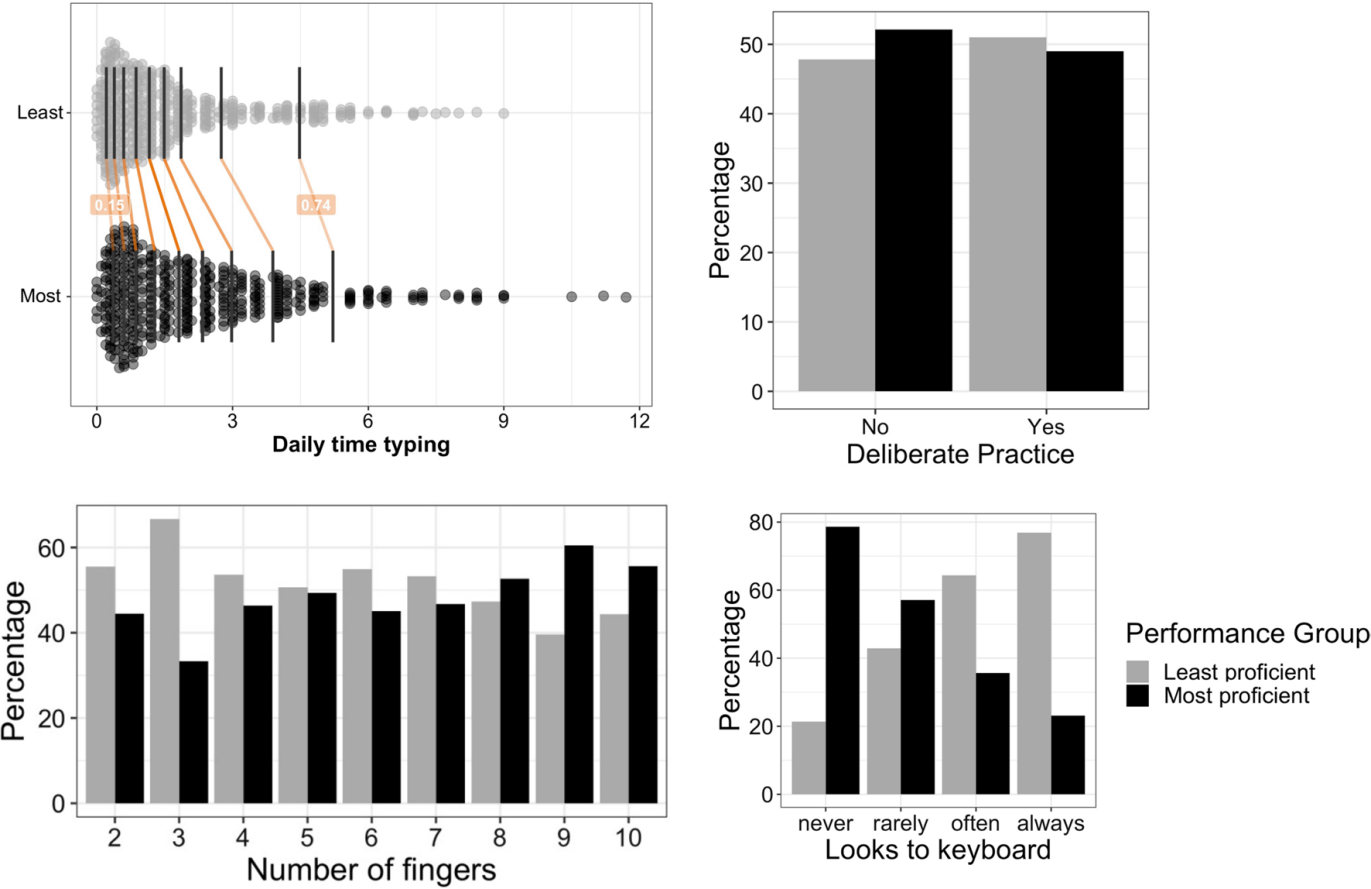
Typing habits according to performance groups. From top to bottom and left to right: daily time spent typing, deliberate practice, number of fingers used for typing, frequency of looks to the keyboard while typing. For the bar graphs, the percentage of most and least proficient typists within each level of the factor of interest is plotted

Our first research question asked whether typing habits (i.e. time spent typing a day, number of fingers used to type, etc.) would vary according to the performance groups defined in the previous section. Our hypothesis was that the two performance groups would display different typing practices, thus establishing a link between performance achievement and specific typing habits. Our primary expectations were that most proficient typists would in general spend more time typing than least proficient typists, and that there would be more individuals among proficient typists that report deliberate practice (i.e. engaging in an effortful practice to optimize performance). Fitting a linear regression model for each variable revealed that most proficient typists reported significantly more years of practice (*β* = 0.660, *t* = 2.52, *p* = 0.012), more time spent typing per day (*β* = 0.385, *t* = 5.44, *p* < 0.001), less lecture note-taking by handwriting (*β* = − 0.93, *z* = − 6.44, *p* < 0.001), more fingers used when typing (*β* = 0.40, *t* = 3.1, *p* = 0.0021), and fewer looks at the keyboard when typing (*β* = − 1.31, *z* = − 9.44, *p* < 0.001). Notably, in our dataset, performance group was not significantly associated with the report of deliberate practice in order to improve typing performance (*β* = 0.055, *z* = 0.39, *p* = 0.70; see Table 2 and Fig. 5). The result tables of the full models can be found in Additional file 1: Appendix 5.

**Table 2:** Characteristics of performance groups on typing habits variables

	Least proficient	Most proficient	Significance level
Daily time spent typing (hours)	1.7 ± 1.7	2.4 ± 2.1	< 0.001
Years of practice	9.2 ± 4.7	10.3 ± 4.3	0.0119
Number of fingers used	6.9 ± 1.9	7.3 ± 1.9	0.0021
*Deliberate practice (N)*			
Yes	277	266	0.700
No	155	169	
*Looking to keyboard (N)*			
Never	22	81	< 0.001
Rarely	176	234	
Often	191	106	
Always	40	12	
*Lecture note-taking (N)*			
Hand	292	200	< 0.001
Keyboard	139	235	

Values are either counts of participants (N) or averages and standard deviations across participants

### Do the experimental manipulations that are known to reliably impact professional touch-typists exhibit a differential effect according to performance group?

Variables that are known to reliably impact professional typing performance are bigram frequency, bimanual transition, and word length. The following analysis will focus on these, aiming to answer the following questions: (1) Do these variables have an effect in our population's performance? (2) Are the effects of these variables different among most and least proficient typists?

**Fig. 6:**
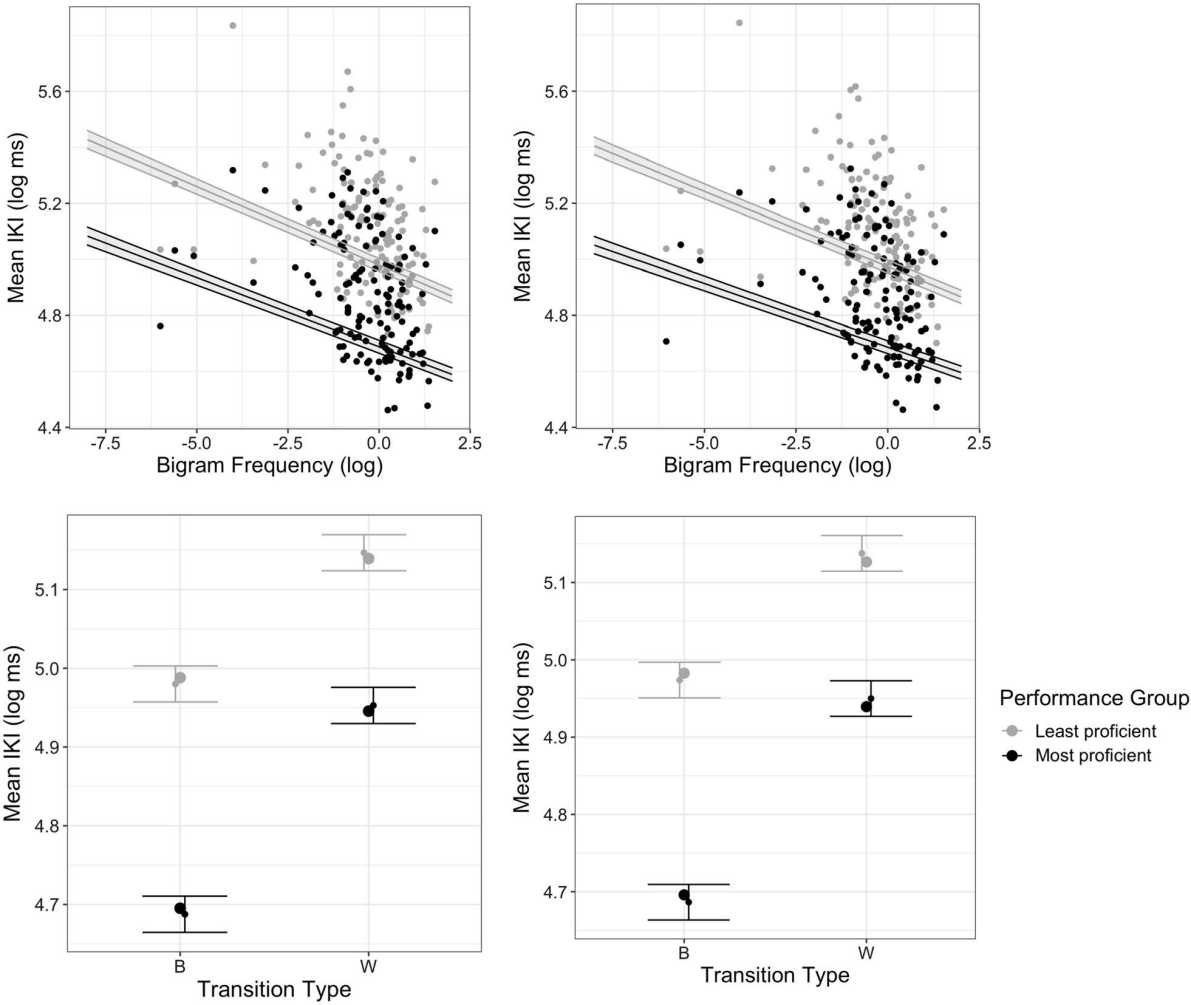
Effect of bigram frequency (top) and transition type (bottom) on IKIs in picture naming (left) and word copying (right) tasks. IKIs are plotted in log scale, as they were entered in the model. Predicted effects from the mixed model analysis are plotted on top of the data with 95% confidence intervals (obtained from ggeffects package (Lüdecke, 2018)). Bigram frequency is centred and scaled

**IKIs**. Individual IKIs were submitted to a mixed-effect linear regression analysis including performance group (defined above) as a factor, as well as the main effects and interactions with performance group of various linguistic and motoric variables as predictors: bigram frequency, transition type, length, position of the IKI in the word, and word frequency. Both tasks showed very similar effects, in direction and magnitude, and will be discussed jointly. The analysis revealed that the main effect of performance group was significant (picture naming task: *ß* = − 0.29, *t* = − 22.8, *p* < 0.001, word copying task: *ß* = − 0.29, *t* = − 22.7, *p* < 0.001; Table 3, Fig. 6), with most proficient typists showing lower IKIs than least proficient typists. The variables of interest outlined above, bigram frequency, transition type, and word length, also yielded significant main effects. Bimanual intervals, frequent bigrams, and shorter words had lower IKIs than unimanual, less frequent bigrams, or long words. Importantly, all three factors significantly interacted with performance group. The effect of transition type was larger for most than least proficient typists. However, the effect of bigram frequency and word length was stronger for least than most proficient typists.

**Table 3:** Results of mixed-effect model on IKI for the picture naming and word copying tasks

	Picture naming				Word copying			
	*ß*	SE	*t*	*p*	*ß*	SE	*t*	*p*
(Intercept)	4.98	0.012	426.45	< 0.001***	4.97	0.012	422.76	< 0.001***
*Variables of interest*								
Performance group	− 0.29	0.013	− 22.82	< 0.001***	− 0.29	0.013	− 22.72	< 0.001***
Transition type	0.17	0.0028	58.83	< 0.001	0.16	0.0027	61.03	< 0.001***
Transition type × performance group	0.098	0.0035	27.82	< 0.001***	0.100	0.0033	29.91	< 0.001***
Bigram frequency (log)	− 0.056	0.0015	− 38.54	< 0.001	− 0.054	0.0014	− 39.15	< 0.001***
Bigram frequency × performance group	0.0065	0.0018	3.60	< 0.001***	0.0083	0.0017	4.88	< 0.001***
Length	0.029	0.0073	3.94	< 0.001***	0.031	0.0075	4.14	< 0.001***
Length × performance group	− 0.016	0.002	− 8.44	< 0.001***	− 0.016	0.0018	− 8.75	< 0.001***
*Control variables*								
Position	− 0.044	0.0014	− 31.72	< 0.001***	− 0.043	0.0013	− 33.17	< 0.001***
Position × performance group	− 0.0069	0.0019	− 3.60	< 0.001***	− 0.0053	0.0018	− 2.94	0.0033**
Word frequency (log)	− 0.037	0.0075	− 4.91	< 0.001***	− 0.038	0.0076	− 4.93	< 0.001***
Word Frequency × performance group	− 0.0015	0.0018	− 0.81	0.418	0.0013	0.0017	0.79	0.431
Trial	0.0041	0.0013	3.26	0.0011**	0.0070	0.0012	5.84	< 0.001***
Trial × performance group	− 0.0018	0.0018	− 0.99	0.322	− 0.00070	0.0017	− 0.42	0.672

Other variables such as the position of the IKI within the word also yielded a significant main effect and an interaction with performance group, with later positions being associated with shorter IKIs, an effect that was stronger in proficient typists. Notably, word frequency revealed a significant main effect (such that more frequent words are typed faster) that did not interact with performance group.

In sum, the observed main effect of the classically reported variables (e.g. bigram frequency, transition type) on IKIs confirmed that they did have a significant effect on our population; their significant interactions with performance groups suggested that while effects were present for both groups, most proficient typists tended to display stronger effects than least proficient typists.

**Fig. 7:**
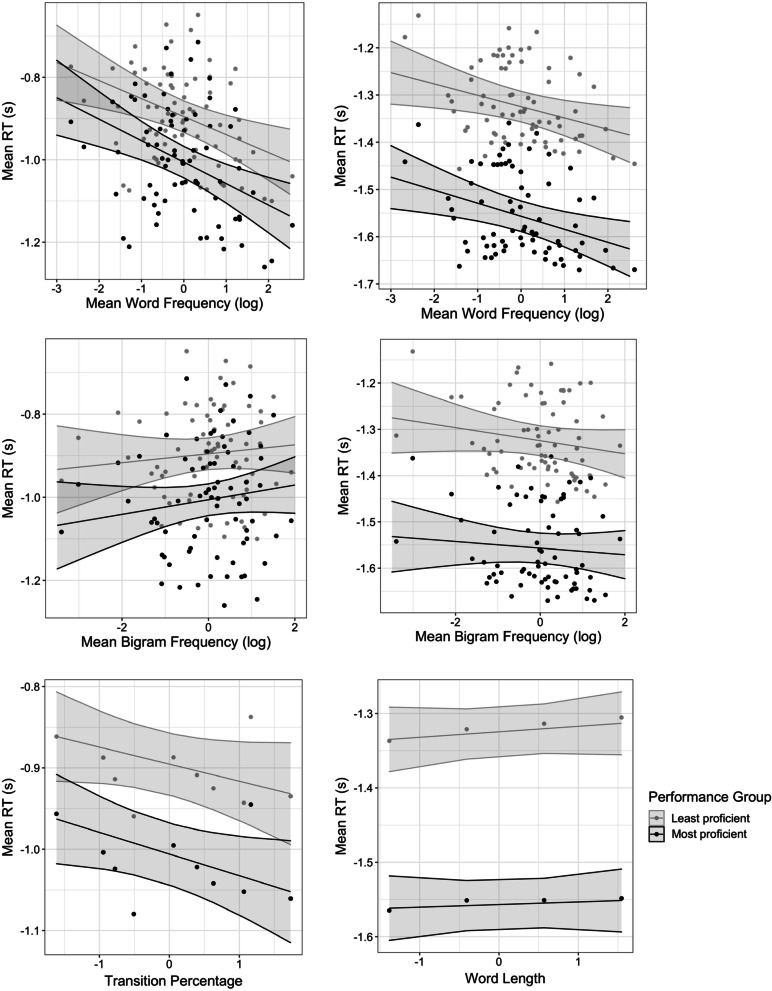
Effect of word frequency (top), mean bigram frequency (middle) and transition percentage and length (bottom) on RT in picture naming (left) and word copying (right) tasks. RT is plotted in a negative inverse scale, as it was entered in the model. Predicted effects from the mixed model analysis are plotted on top with 95% confidence intervals (obtained from ggeffects package). Continuous predictors are centred and scaled

**RTs.** RTs were also submitted to a mixed-effect linear regression analysis that included performance groups as a factor, and the main effects and interactions with performance group of the same various linguistic and motoric variables as predictors: bigram frequency, transition percentage, word frequency, word length, and laterality of the first keystroke. In contrast with the results observed with IKIs, there were some differences in terms of direction, magnitude, and significance of the effects between both tasks (see Table 4, Fig. 7). Performance group again had a significant main effect in both tasks (picture naming task: *ß* = − 0.11, *t* = − 13.4, *p* < 0.001, word copying task: *ß* = − 0.23, *t* = − 16.6, *p* < 0.001), with most proficient typists showing lower RTs than least proficient typists. The variables of primary interest outlined above for IKIs, bigram frequency and transition type, did not show a significant main effect in either task. However, transition type did interact significantly with performance group, in the picture naming task only: the facilitative effect of transition type was observed for most but not for least proficient typists. Bigram frequency interacted significantly with performance group in both tasks, and revealed effects in opposite directions across tasks. In picture naming, bigram frequency had an inhibitory effect, stronger for most than least proficient typists. In word copying, bigram frequency had a facilitatory effect, stronger for least than most proficient typists. Word length did not display a significant main effect on RTs on either task, however, it interacted with performance group in word copying, such that the effect was stronger for least than most proficient typists. Finally, and as expected, word frequency significantly sped up RTs in both tasks. It also significantly interacted with performance group, with most proficient typists being more sensitive than least proficient.

**Table 4:** Results of mixed-effect model on RT for the picture naming and word copying tasks

	Picture naming				word copying			
	*ß*	SE	*t*	*p*	*ß*	SE	*t*	*p*
(Intercept)	− 0.895	0.0194	− 46.188	< 0.001***	− 1.325	0.0165	− 80.535	< 0.001***
*Variables of interest*								
Performance group	− 0.11	0.0082	− 13.427	< 0.001***	− 0.23	0.0140	− 16.55	< 0.001***
Word frequency (log)	− 0.044	0.0141	− 3.104	0.0028**	− 0.024	0.00988	− 2.43	0.018*
Word frequency × performance group	− 0.0084	0.0017	− 4.898	< 0.001***	− 0.0036	0.00150	− 2.41	0.016*
Bigram Frequency (log)	0.011	0.0143	0.752	0.455	− 0.014	0.0102	− 1.39	0.170
Bigram frequency × performance group	0.0069	0.0018	3.922	< 0.001***	0.0071	0.00153	4.62	< 0.001***
Length	0.012	0.0139	0.89	0.377	0.0074	0.00985	0.749	0.457
Length × performance group	0.00078	0.0017	0.453	0.651	− 0.0039	0.00149	− 2.60	0.0095**
*Control variables*								
Transition percentage	− 0.021	0.0136	− 1.538	0.129	0.0022	0.00976	0.221	0.826
Transition percentage × performance group	− 0.0057	0.0017	− 3.358	< 0.001***	− 0.00029	0.00147	− 0.197	0.844
Laterality (R-)	− 0.0067	0.0272	− 0.246	0.806	− 0.0038	0.0193	− 0.198	0.844
Laterality × performance group	− 0.000029	0.0033	− 0.009	0.993	0.0030	0.00292	1.04	0.298
Trial	0.012	0.0012	10.41	< 0.001***	− 0.0060	0.00104	− 5.78	< 0.001***
Trial × performance group	0.0037	0.0017	2.214	0.027*	0.00041	0.00144	0.282	0.778

Performance group were coded as treatment contrasts

In sum, none of the variables classically reported on IKIs had a significant main effect on RTs in our population, although some significant interactions with performance groups suggested that they had contrasting effects in most and least proficient typists.

**Complementary exploratory analyses.** We ran two complementary analyses not initially planned in the preregistration. Each of these analyses involved a different definition of the participant groups. In both cases, we re-evaluated the effects of group on typing habits and the combined effects of group and of stimuli characteristics in the picture naming and copy-typing tasks.

In the first complementary analysis, we created performance groups based on a split of the initial distribution in fifths instead of thirds. We ran the same statistical models as in the main analyses but we considered only the first and last fifths of the distribution, to target more contrasted performance groups, comprising 260 participants each. The main effects observed for the analysis of typing habits and performance groups were similar. In particular, even with this more stringent split, the effect of group on the amount of deliberate practice remained non-significant, while the differences in terms of looking at hands and number of fingers used remained significant. The main effects of stimuli characteristics in the picture naming and copy-typing tasks were also observed with this more stringent group split. On IKIs, effects were similar, in the same direction, in the same range, and presented the same significance (except for trial on picture naming and word frequency for word copying).

In the second complementary analysis, we considered the possible impact of typing style, a variable that has been strongly associated with typing performance (Dhakal et al., 2018; Feit et al., 2016). Based on a reviewer’s suggestion, we defined the two groups based on the distribution of self-reported number of fingers as a proxy for typing style. We compared two groups, one of participants that used 6 fingers or less (*N* = 522) and the other that used 8 or more fingers (*N* = 569). Again, we excluded the middle category (the 210 participants that reported using 7 fingers) to avoid overlap between groups. We observed a significant but weak correlation between self-reported number of fingers and typing speed, *r* = 0.157, *p* < 0.001, consistent with Dhakal et al. (2018) where the correlation between typing speed and self-reported number of fingers was *r* = 0.38. It is important to note that the distribution of number of fingers used in Dhakal et al. (2018) was different from ours: the highest proportion of their population (47%) reported using 9–10 fingers, while the majority of our typists (40%) reported using between 6 and 8 fingers.

The group split as a function of typing style led to effects on typing practice and habits that were fully consistent with the effects found with performance groups: typing style groups differed in their daily time spent typing, frequency of looks to the keyboard while typing, years of practice and amount of lecture note-taking by handwriting, but not in deliberate practice.

As expected, the typing style had a main effect on performance in the two tasks. The main effects of stimuli characteristics in the picture naming and copy-typing tasks were also similar when groups were split based on typing style. The only exception was the interaction of finger group with transition type (picture naming: *ß* = − 0.018, *t* = − 4.74, *p* < 0.001, word copying: *ß* = − 0.010, *t* = − 2.88, *p* = 0.004) and bigram frequency (picture naming: *ß* = − 0.01, *t* = − 4.94, *p* < 0.001, word copying: *ß* = − 0.012, *t* = − 6.36, *p* < 0.001) on IKIs, which were also significant but of opposite direction compared to the model with performance groups. Typists who use more fingers show a stronger effect of bigram frequency, but a smaller effect of transition type than those who use less fingers. In sum, the predicted effect of bimanual transition was found when groups were split as a function of performance, while the predicted effect of bigram frequency was found when groups were split as a function of finger use. This dissociation between typing style and performance fits with the complexification of typing skills nowadays. Combining the two characteristics could be interesting in future investigations of typing expertise, but a more accurate assessment of typing style (for example, using motion tracking methods, Feit et al., 2016) is essential to guarantee a completely reliable analysis. The full report of the complementary results can be found in Additional file 1: Appendix 7 and 8.

## Discussion

This study aimed to link typing performance to typing practice and habits and to variables known to index cognitive and motor processes. The results show that using a keyboard frequently (years of practice, daily time of practice, note-taking) can, by itself, lead to the development of highly proficient skills associated with the use of more fingers and less looks at hands, even without deliberate practice. When assessing the effects of cognitive and motor factors, we found that most factors affected the IKIs of both groups, but to different extents. RTs, in contrast, were generally less affected by cognitive and motor factors, but their interactions with performance group differed according to the task (word copying and picture naming).

### Linking typing habits to performance groups

We observed that deliberate practice did not significantly differ between performance groups. This complements Dhakal et al.’s (2018) observation of a small difference in typing speed between formally trained and untrained typists in their sample. Deliberate practice has been hypothesized to be crucial to reach high levels of expertise (Ericsson et al., 1993), but its effects are increasingly debated (Ericsson & Harwell, 2019; Hambrick et al., 2020; Macnamara et al., 2014). Other types of practice, such as “naïve” practice (“just doing something repeatedly, and expecting that the repetition alone will improve one’s performance”; Ericsson, 2016), could be as or perhaps even more efficient. In the case of typing, incidental learning may occur through repeated exposures to the keyboard without any intentional objective of improvement (Feit et al., 2016; Grabowski, 2008). In terms of mechanisms, Behmer and Crump (2016) argued that typing acquisition and performance are supported by general learning and memory processes which are at play when sequential information is repeatedly produced (see also Cleeremans, 1993; Logan, 1988). These processes, which rely upon the statistical structure of the material being typed and the configuration of the keyboard, may not be specifically potentialized by deliberate practice. In that perspective, irrespective of practice being deliberate or naïve, an important determinant of performance would rather be the amount of accumulated practice, in agreement with studies of other types of expertise (Ericsson et al., 1993; Keith & Ericsson, 2007). In support of this view, the most and least proficient typists of our sample did differ in their amount of daily practice and in their age and number of years of practice.

Even for the least proficient typists, the amount of reported daily practice (1.7 h on average) is arguably huge. It is comparable to the typical amount of practice of professional typists enrolled in twentieth century studies (11 h per week; Salthouse, 1984). In the most proficient group, the amount of weekly practice could be compared to the time devoted by elite athletes or professional musicians to their respective skills. And this amount does not include the time spent typing on a smartphone, where similar inter-manual coordination and memorization processes may apply and reinforce typing skills (Cerni et al., 2016a, 2016b). In short, the amount of practice that typists are getting nowadays through the simple use of tools that have keyboards (computers, smartphones) seems to have rendered the voluntary act of practising largely irrelevant.

The least and most proficient typists also differ on how they achieve such performance, in particular in terms of how many fingers they use to type, and whether they tend to type without looking at their fingers. The number of fingers used has been shown to strongly affect typing performance (Dhakal et al., 2018) and the automation of typing skills (Logan et al., 2016). Our results clearly support this view, but they also show that the distributions of finger use in the two groups overlaps substantially. This is in agreement with data indicating that variable strategies of finger use can lead to similar performance levels (Feit et al., 2016; Logan et al., 2016). The most discriminant difference was the more frequent use of 3 fingers in low performers, and the more frequent use of 9 fingers in high performers. For the intermediate values of finger use, the frequency values were similar for the two groups. In fact, in terms of practice factors, the biggest difference we observed between the groups is in the look at hands while typing (Fig. 4). Although reliance on visual feedback from the hands is probably the less studied factor in experimental investigations of typing skills (presumably because of the added complexity of co-registering gaze with typing performance), it may be a relevant index of greater automatization in the high performers. In experts, vision can be important to monitor the hands in order to inhibit inappropriate keystrokes in particular when typing in unusual conditions (Tapp & Logan, 2011). However, it is likely that under normal conditions, with higher typing skills, proprioceptive feedback becomes the dominant feedback source to control the sequence, while vision becomes devoted to monitor the outer loop (Crump & Logan, 2010b; Logan & Crump, 2011; Salthouse, 1986). In short, we point to two practice factors—finger use and looks to the keyboard—that were associated with proficiency, possibly because they index the level of automatization of typing (Logan & Crump, 2011, Fig. 2) and the efficiency of motor programming.

These differences observed in typing habits could lead to differences in the underlying cognitive architecture of the two performance groups. If so, experimental variables previously identified for their influence on touch-typing performance should affect the most proficient typists of our sample more strongly than the least proficient typists (Fig. 2).

### Assessing the impact of experimental manipulations known to reliably impact professional touch-typing across performance groups

Our investigation of the effect of stimuli variables on the two performance groups replicated in the current population some of the classical effects previously reported in twentieth century typists. Factors linked to peripheral processes (bigram frequency and transition type; Coover, 1923; Gentner et al., 1988; Grudin & Larochelle, 1982; Kinkead, 1975; Larochelle, 1983; Terzuolo & Viviani, 1980) had significant effects on IKIs and differed in magnitude across the two performance groups. The *reduced* effect of transition type in less proficient typists may have two origins: less automatized parallel processing and less systematic finger-key mapping such that the transition factor less faithfully describes the actual gestures. As expected, the least proficient group was the only group to display an effect of length (Gentner, 1983b; Larochelle, 1983; Sternberg et al., 1978). However, this effect was limited to the copy-typing task. This selective task-specific effect could be related to processing of the visual input and the length of the character string in the copy-typing task. It is not necessarily in contradiction with previous literature since early studies used transcription typing and did not test for the generalizability of length effects to other typing tasks. We also note that length effects in picture naming have proved to be elusive in the oral modality (Alario et al., 2004; Meyer et al., 2003). Finally, as typically observed in language production tasks, lexical frequency had a facilitatory effect on RTs (Baus et al., 2013; Inhoff, 1991; Pinet et al., 2016).

It is important to point out that even if the factors linked to peripheral processes (transition percentage and bigram frequency) yielded the expected observations for the most proficient individuals, they were also present in least proficient typists, which is evidenced by significant effects with both subgroups. Moreover, bigram frequency effects were stronger in the low-performance group. This result was unexpected because Behmer and Crump (2016) reported increased sensitivity to bigrams with increasing typing speed. Keeping in mind the relatively good skill level and the rather extensive amount of practice even in the least proficient group, it is likely that statistical regularities of the sequences typed have been firmly integrated in both groups. We know that low proficient typists are more likely to use variable finger-to-key mappings (Feit et al., 2016), which might lead to adaptations to type frequent bigrams, for instance, more often with hand alternations than a 10-finger typist would. This could improve the performance for such frequent bigrams and lead to stronger frequency effects in least proficient typists. In relation to this, typing style as indexed by both the number of fingers and typing strategies (Feit et al., 2016) could be complementary to typing proficiency in future studies aiming to assess the effects of experimental manipulations on typing expertise. Finding motor effects in the least proficient typists, sometimes even stronger than in the most proficient typists, suggests that the established model of typing expertise (see Fig. 2) that separates novice and expert typists may not be entirely accurate to describe our sample of typists.

We also observed several effects that could challenge the basic assumption whereby typing expertise leads to the implementation of automated motor routines, while language (orthographic, semantic, etc.) processes remain unaffected. In keeping with this assumption, we predicted that expertise would exert its influence mainly by automatizing typing execution through the inner loop (Fig. 2), leading to interactions with proficiency on typing execution, measured by IKIs. However, we also report several interactions with performance groups on RTs. These interactions, when present in the two tasks, could also have a motor origin, as motor programming contributes to the reaction time in typing (Logan & Crump, 2011). However, when the interactions are different between the two tasks, it rather suggests that task-specific cognitive processes are affected by typing proficiency. For instance, bigram frequency interacted with performance group but had opposite effects across the two tasks. This could indicate that bigram frequency affects cognitive processes that are task-specific and does not affect these processes in the same way in low- and high-performance typists. The same type of observation was made for the selective effect of length seen in least proficient typists in the copy task or for the stronger effect of word frequency for the most proficient typists.

Conversely, motor variables such as transition percentage had a facilitatory effect on the RTs of the most proficient typists, only in the picture naming task. Previous work indicates that strong relationships between perceptual/cognitive and motor representations of letters and letter sequences can develop in skilled typists (Beilock & Holt, 2007; Cerni et al., 2016a, 2016b; Rieger, 2004, 2007; Van den Bergh et al., 1990). For instance, the seminal work of Rieger established that the mere visual perception of a letter automatically primes the corresponding finger movements in skilled typists (Rieger, 2004, 2007). Cerni et al. (Cerni et al., 2016a, 2016b) showed that, at the word level, performance in tasks such as lexical decision can be affected by typing expertise.

In sum, intensive practice of typing might influence processes upstream from motor programming and execution, putting into question the so-far assumed cognitive architecture of typing skills (Fig. 2). While our findings point in this direction, more work is needed to confirm these claims.

### Habits and cognitive processes underlying typing performance

As noted in the introduction, we aimed at combining two previous approaches, which focused on characterizing the habits associated with proficient typing (Keith & Ericsson, 2007), and on identifying the cognitive processes underlying proficiency (Behmer & Crump, 2016). Our results revealed that the most expected factor, i.e. deliberate practice, did not significantly affect typing proficiency within our sample. On the other hand, least proficient typists showed similar effects (although smaller in scale) than most proficient typists on some peripheral factors, and performance interacted with upstream cognitive factors in both groups. Our combined approach allows us to conclude that differences in the underlying cognitive processes in our large sample of university students might be more quantitative than qualitative as practice and proficiency increases (Fig. 2), with the cognitive architecture previously defined for expert being widespread across the whole student population.

### Implications and limitations

On top of challenging current theories of expertise in typing, the current results have important practical implications for experimental studies involving typists. Despite some previous description of the current distribution of typing skills (Dhakal et al., 2018; Feit et al., 2016), selection criteria for so-called expert typists were chosen somewhat arbitrarily (e.g. typing speed above a somewhat arbitrary threshold, requirement to practice touch-typing without looking at the keyboard, etc. (Logan & Zbrodoff, 1998)). Our description and available dataset of the skill distribution in the population of young students will be a relevant tool for researchers in cognitive science interested in language or motor sequence production (Baus et al., 2013; Pinet et al., 2016; Scaltritti et al., 2017), complementing other large database studies (Dhakal et al., 2018). Our results can also provide a benchmark for the clinical assessment of typing skills which might become relevant in the coming years for young dysgraphic patients. Finally, this research also has the potential to inform issues in education research in relation to the increasing role of typed written production and computerized tools in educational settings (Grabowski, 2008).

One limitation of our study is a potential bias in the recruitment of participants for an online experiment. Perhaps the general topic of the study prompted skilled typists to volunteer more because they felt quite confident on their abilities. Another possible limitation is the assessment of typing habits through self-reported questionnaires, which may not be as efficient as direct interviews, such as those implemented by Keith and Ericsson (2007) and others. In addition, we did not try to measure the consistency of the keystroke/finger mappings (as did Logan et al., 2016), and the amount of practice was estimated by the participants based on one single question. Given the present results, it would be important in the future to refine the estimation of relevant practice factors and their effect on the development of typing skills. Of course, there will be trade-off between the ability to collect larger amounts of data online and the better control of the interview procedures during in-person experiments.

## Conclusion

Coupling the investigation of practice and cognitive factors on typing performance can help better understanding typing skills in the current population. Our data indicate that incidental learning through experience (so-called naïve practice) can lead to a continuous distribution of typing skills in a large population. Practice frequency estimates reveal the massive use of computer keyboards, compared to other tools in other domains of expertise (e.g. music instruments or sports gear). The expected effects of the classically reported variables on typing performance are present in our sample (e.g. bigram frequency, transition type). Although effects were generally stronger with higher proficiency, they were also evident in the least proficient typists. This indicates that, in a vast majority of university students, the cognitive processes enabling typing are likely those of experts*.* In addition, some experimental effects suggested that the degree of automatization of typing skills may modify the cognitive architecture underpinning the task (i.e. the separation between word retrieval and typing execution). Overall, our findings challenge the applicability of standing models of typing expertise to the current generation of young typists.

## Supplementary Information

**Additional file 1**. Supplementary material and analyses.

## Data Availability

Data and material are available at the following repository: https://osf.io/v92fy/?view_only=87885752038b4be190d532143fdedb07.

## Footnotes

1 “Formal training” here refers to having taken typing lessons to follow the standard mapping. This is not what we refer later as “deliberate practice”, which should be understood as having tried to improve one’s way of typing (e.g. through free online training), mostly to increase typing speed but not necessarily to change finger-to-key mappings.
