# Correction: Typing expertise in a large student population

**DOI:** 10.1186/s41235-022-00446-x

**Published:** 2022-10-26

**Authors:** Svetlana Pinet, Christelle Zielinski, F.-Xavier Alario, Marieke Longcamp

**Affiliations:** 1 BCBL, Basque Center On Cognition, Brain and Language, Paseo Mikeletegi 69, 2º, 20009 Donostia-San Sebastián, Spain; 2 Basque Foundation for Science, Bilbao, Spain; 3 ILCB - Institute of Language, Communication and the Brain, Aix Marseille Univ, Aix-en-Provence, France; 4 CNRS, LPC, Aix Marseille Univ, Marseille, France

## Correction to: Cognitive Research: Principles and Implications (2022) 7:77 10.1186/s41235-022-00424-3

The original article (Alario et al., 2004) mistakenly omitted co-last authorship between F.-Xavier Alario and Marieke Longcamp which has since been re-instated.
