# Supplementary material for: Typing expertise in a large student population
Source: Cogn Res Princ Implic. 2022 Aug 5;7:77. doi: 10.1186/s41235-022-00424-3 (PMC9356123; doi:10.1186/s41235-022-00424-3)
Supplement: Supplementary file 1 — Additional file 1. Supplementary material and analyses. [file 41235_2022_424_MOESM1_ESM.docx]

**Appendix 1: Material**

There were **84 pictures** for the picture naming task and the corresponding **84 words** for the copying task (for translations and psycholinguistic properties, see Table S1).

*Table S1: Psycholinguistic properties of the items used in the copying and picture naming tasks. Picture and linguistic properties from databases Alario & Ferrand, 1999; Bonin et al., 2003; or used in Pinet et al., 2015; AoA: Age of acquisition (from above databases); Freq: lexical frequency (freqfilms2 from lexique.org); Lat. 1st: laterality (L: left, R: right) of the first letter of the word; Lat. transitions: percentage of bimanual transitions.*

| Item | English | Database | Length | AoA | Freq | Lat. 1st | Lat. transitions |
| --- | --- | --- | --- | --- | --- | --- | --- |
| ananas | pineapple | Alario | 6 | 2.46 | 2 | L | 80% |
| ancre | anchor | Alario | 5 | 3.32 | 4.6 | L | 50% |
| balai | broom | Alario | 5 | 1.95 | 8.2 | R | 100% |
| banane | banana | Alario | 6 | 1.58 | 6.1 | R | 100% |
| banc | bench | Alario | 4 | 2.15 | 9 | R | 100% |
| bonbon | candy | Pinet | 6 | NA | 6.9 | R | 0% |
| bougie | candle | Alario | 6 | 1.96 | 7.4 | R | 60% |
| briquet | lighter | Bonin | 7 | 3.2 | 10 | R | 83% |
| bulle | bubble | Bonin | 5 | 1.75 | 3 | R | 25% |
| cadeau | gift | Bonin | 6 | 1.35 | 98.1 | L | 20% |
| cage | cage | Alario | 4 | 2.27 | 16.6 | L | 0% |
| carafe | carafe | Pinet | 6 | NA | 1 | L | 0% |
| carotte | carrot | Alario | 7 | 1.58 | 2.5 | L | 33% |
| carte | playing card | Bonin | 5 | 2.3 | 96.1 | L | 0% |
| cerf | deer | Pinet | 4 | NA | 6.2 | L | 0% |
| chaise | chair | Alario | 6 | 1.38 | 32.7 | L | 80% |
| chapeau | hat | Alario | 7 | 1.62 | 48.6 | L | 83% |
| chat | cat | Alario | 4 | 1.35 | 57.7 | L | 67% |
| chemise | shirt | Alario | 7 | 2.04 | 36.5 | L | 67% |
| cheval | horse | Alario | 6 | 1.54 | 85.4 | L | 60% |
| citron | lemon | Alario | 6 | 1.88 | 8.1 | L | 60% |
| coeur | heart | Alario | 5 | 1.81 | 225 | L | 100% |
| corne | horn | Bonin | 5 | 2.3 | 2.6 | L | 100% |
| couteau | knife | Alario | 7 | 1.65 | 51.1 | L | 50% |
| crabe | crab | Alario | 5 | 2.38 | 4.9 | L | 50% |
| cravate | tie | Alario | 7 | 2.38 | 16 | L | 0% |
| cube | cube | Bonin | 4 | 1.8 | 1.6 | L | 67% |
| cuisine | kitchen | Bonin | 7 | 1.55 | 85.1 | L | 67% |
| domino | domino | Bonin | 6 | 2.55 | 0.4 | L | 20% |
| drapeau | flag | Alario | 7 | 2.58 | 14.7 | L | 50% |
| gant | glove | Alario | 4 | 2 | 9.9 | L | 67% |
| garage | garage | Pinet | 6 | NA | 24.4 | L | 0% |
| gare | station | Pinet | 4 | NA | 40.3 | L | 0% |
| hibou | owl | Pinet | 5 | NA | 4.1 | R | 0% |
| igloo | igloo | Alario | 5 | 3.08 | 0.5 | R | 50% |
| indien | native american | Bonin | 6 | 2.35 | 4.1 | R | 80% |
| journal | newspaper | Bonin | 7 | 2.55 | 72.5 | R | 67% |
| jupe | skirt | Alario | 4 | 1.65 | 10.1 | R | 33% |
| lapin | rabbit | Alario | 5 | 1.65 | 26.6 | R | 50% |
| licorne | unicorn | Bonin | 7 | 2.75 | 1.4 | R | 83% |
| lime | lime | Alario | 4 | 3.19 | 1.5 | R | 33% |
| lion | lion | Alario | 4 | 1.69 | 14.6 | R | 0% |
| loup | wolf | Pinet | 4 | NA | 21 | R | 0% |
| main | hand | Alario | 4 | 1.12 | 286.6 | R | 67% |
| maison | house | Alario | 6 | 1.38 | 570.3 | R | 80% |
| marin | sailor | Bonin | 5 | 2.8 | 8.5 | R | 50% |
| micro | microphone | Bonin | 5 | 2.6 | 11.3 | R | 50% |
| montre | watch | Alario | 6 | 2.19 | 43.9 | R | 20% |
| moto | motorbike | Alario | 4 | 2.23 | 22.6 | R | 67% |
| mouche | fly | Alario | 6 | 1.77 | 15.4 | R | 60% |
| oeil | eye | Alario | 4 | 1.38 | 97.1 | R | 67% |
| ours | bear | Alario | 4 | 1.62 | 24 | R | 33% |
| palmier | palmtree | Alario | 7 | 3.19 | 1.7 | R | 50% |
| peigne | comb | Alario | 6 | 2 | 6.1 | R | 100% |
| piano | piano | Alario | 5 | 2 | 21.5 | R | 50% |
| pied | foot | Alario | 4 | 1.31 | 105.5 | R | 33% |
| pion | pawn | Pinet | 4 | NA | 3 | R | 0% |
| piscine | swimming pool | Bonin | 7 | 2.05 | 22.2 | R | 50% |
| plante | plant | Bonin | 6 | 2.4 | 9 | R | 60% |
| plume | feather | Alario | 5 | 2.16 | 6.5 | R | 25% |
| poire | pear | Alario | 5 | 1.81 | 5.7 | R | 25% |
| poisson | fish | Alario | 7 | 1.62 | 53.6 | R | 33% |
| poulet | chicken | Bonin | 6 | 2 | 32.3 | R | 20% |
| puits | well | Alario | 5 | 2.77 | 19.5 | R | 25% |
| puzzle | jigsaw puzzle | Bonin | 6 | 2.3 | 4.4 | R | 60% |
| robot | robot | Bonin | 5 | 2.05 | 15 | L | 50% |
| rose | rose | Bonin | 4 | 2.1 | 11.1 | L | 67% |
| sabot | shoe | Bonin | 5 | 2.55 | 1.8 | L | 50% |
| selle | saddle | Alario | 5 | 3 | 8.9 | L | 50% |
| tambour | drum | Alario | 7 | 2.15 | 7.8 | L | 33% |
| tampon | stamp | Bonin | 6 | 2.85 | 3 | L | 20% |
| tarte | pie | Pinet | 5 | NA | 10.4 | L | 0% |
| tasse | cup | Pinet | 5 | NA | 18.5 | L | 0% |
| tente | tent | Bonin | 5 | 2.65 | 14.4 | L | 50% |
| tigre | tiger | Alario | 5 | 2.31 | 11.1 | L | 50% |
| tortue | turtle | Alario | 6 | 1.92 | 4 | L | 80% |
| vache | cow | Alario | 5 | 1.6 | 36.2 | L | 50% |
| verre | glass | Alario | 5 | 1.23 | 154.1 | L | 0% |
| veste | jacket | Pinet | 5 | NA | 36 | L | 0% |
| volant | steering wheel | Bonin | 6 | 2.55 | 19.2 | L | 80% |


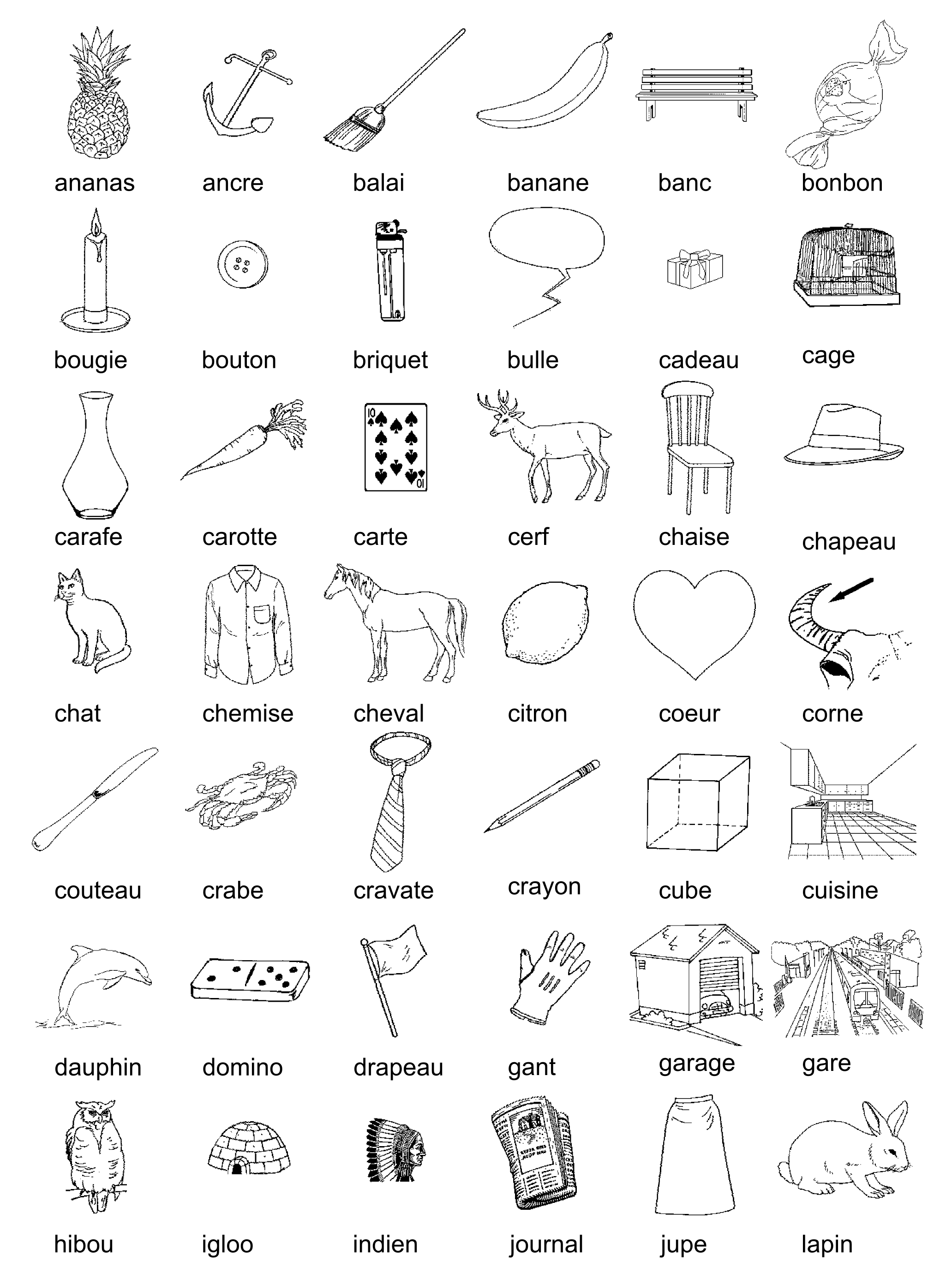

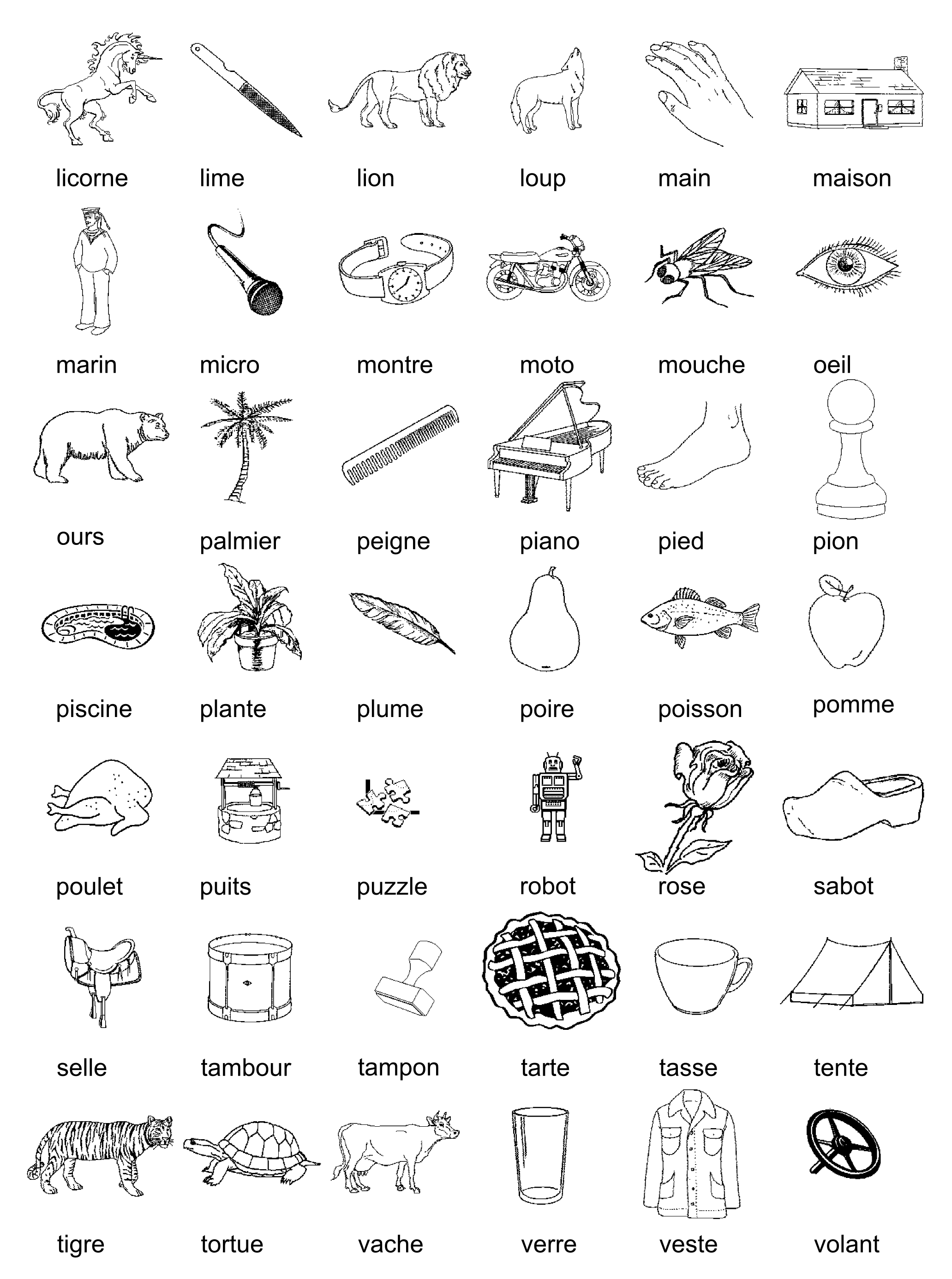


*Figure S1: Images used in the picture naming task.*

*Table S2: Sentences used in the sentence copying task, and their English translations.*

| Sentence number | Original sentence in French | English translation |
| --- | --- | --- |
| Training sentence | La gratification est obligatoire dès lors que le stagiaire est présent dans l'organisme d'accueil. | The gratuity is compulsory as soon as the trainee is present in the host organization. |
| 1 | Vous avez reçu récemment la procédure pour mettre en place votre signature unifiée sur vos courriels. | You recently received the procedure to set up your unified signature on your emails. |
| 2 | Suite à certains problèmes récurrents, nous avons apporté quelques modifications. | Due to some recurring issues, we have made some changes. |
| 3 | Nous vous adressons aussi quelques informations complémentaires pour faciliter l’intégration de votre signature. | We are also sending you some additional information to ease the integration of your signature. |
| 4 | Votre signature est générée automatiquement depuis votre compte personnel dans l’espace numérique de travail. | Your signature is automatically generated from your personal account in the digital workspace. |
| 5 | Une fois la signature générée, vous devez la copier depuis votre compte personnel puis la coller dans votre logiciel de courrier habituel. | Once the signature is generated, you must copy it from your personal account and then paste it into your usual email software. |
| 6 | La signature devient alors modifiable librement. | The signature then becomes freely modifiable. |
| 7 | Si vous rencontrez d’autres problèmes, veuillez vous rapprocher de votre référent technique habituel sur votre site en lui précisant notamment le logiciel utilisé. | If you encounter other problems, please contact your usual technical referent on your site, specifying in particular the software used. |
| 8 | Si le logo proposé ne correspond pas à votre composante, veuillez vous rapprocher de la Direction des Ressources Humaines. | If the proposed logo does not correspond to your school, please contact the Human Resources Department. |
| 9 | Celle-ci pourra mettre à jour vos informations dans le système d’information. | They may update your information in the information system. |

**Exit questionnaire** (translated to English):

Screen 1/7: Some information to finish:

(1.1) The keyboard you just used: azerty, qwerty, azerty modified to qwerty, qwerty modified to azerty

(1.2) Your age:

(1.3) You are: a woman, a man

(1.4) Presumed laterality: left-handed, right-handed, ambidextrous

(1.5) Hand used to write with a pen: left, right

(1.6) Your level of study (or equivalent): Pre-Baccalaureate, Bac, Licence (Bac+3), Master (Bac+5), Doctorate (Bac+8)

(1.7) Are you a student? yes, no

(1.8) In which section? Law, Economics, Management, Humanities, Sciences, Medicine, Pharmacy

(1.9) In which year of study: L1, L2, L3, M1, M2, Doctorate

(1.10) Usual note-taking: on keyboard, by hand

Screen 2/7:

(2.1) Your mother tongue: French, other

(2.2) If other, specify:

(2.3) Are you fluent in (at least) one other language? yes, no

(2.4) Have you ever been followed by a speech therapist for language learning problems? yes, no

(2.5) For what reason(s)? written language, oral language, both, other

(2.6) Are you a musician? yes, no

(2.7) Which instrument(s)?

Screen 3/7: About your keyboard typing (Computer, Tablet):

This part only concerns your use of a computer or tablet (with virtual keyboard), writing on a smartphone will be covered in the next part.

(3.1) Do you write regularly on a keyboard? yes, no

(3.2) On which media(s)? PC, laptop, tablet

(3.3) In total, how much time do you spend on a computer or tablet per day? 0 hour, 1 hour,..., 24 hours

(3.4) What percentage of this time do you spend typing text? 0%, 10%, 20%, ..., 100%

(3.5) What is your main type of activity (maximum 2 choices)? note taking, copying, composing, email, instant chat

(3.6) How did you learn to type on the keyboard? alone, with training

(3.7) For about how many years have you been typing? 1, 2, 3,..., 40 years and over

(3.8) Do you look at your hands while typing? never, rarely, often, always

(3.9) Have you ever tried to significantly improve your typing performance (e.g. by trying to go faster, use more fingers, etc.)? yes, no

(3.10) Do you have any substantial experience other than the QWERTY keyboard (stays abroad, use of another type of keyboard)? yes, no

Screen 4/7:

Select the fingers used to write on the keyboard:


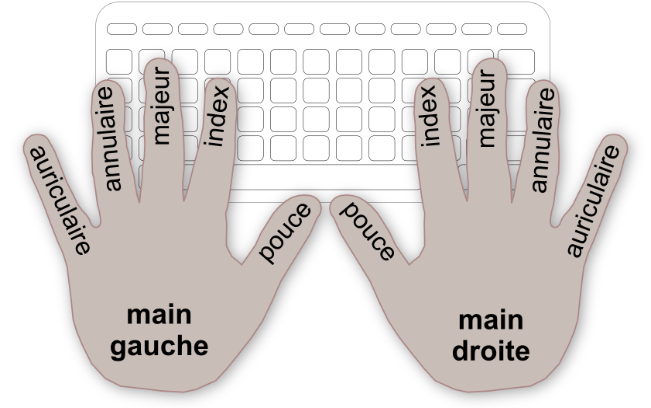


(4.1) Left hand: thumb, index finger, middle finger, ring finger, ring finger, little finger

(4.2) Right hand: thumb, index finger, middle finger, ring finger, ring finger, little finger

Screen 5/7: Practice of writing on another medium (mobile phone, smartphone):

(5.1) In total, how much time per day do you spend writing on another medium (writing time only): 0 minute, 10 minutes, 20 minutes, ..., 1 hour, 2 hours, ..., 24 hours

(5.2) Do you use a smartphone phone? yes, no

(5.3) For how many years? 1, 2, 3, ..., 15 years and over

(5.4) On your phone, do you use a keyboard: QWERTY, other (e. g. number pad)

(5.5) Phonetic writing (e. g. "l8r")? yes, no

Screen 6/7: Handwriting practice:

(6.1) Estimated total handwriting time per day: 0 minute, 10 minutes, 20 minutes, ..., 1 hour, 2 hours, ..., 24 hours

Screen 7/7: And finally:

(7.1) Do you have any comments about your writing practice (digital or handwritten)? yes, no

(7.2) Which ones?

(7.3) Did you have a problem during the online experience? yes, no

(7.4) Which one?

**Appendix 2: Subject exclusion based on task performance.**

Participant exclusion was based on accuracy and length of response (i.e., number of keystrokes) in the picture naming and word copying tasks and exclusion criteria were the following: more than 40% of empty responses, mean length of response below one standard deviation of the mean for each task, mean accuracy below 30% associated with a mean RT below 500ms (see Figure S2). In the sentence copying task, if all sentences produced by the participant had less than half the expected number of characters, this participant was excluded as well.


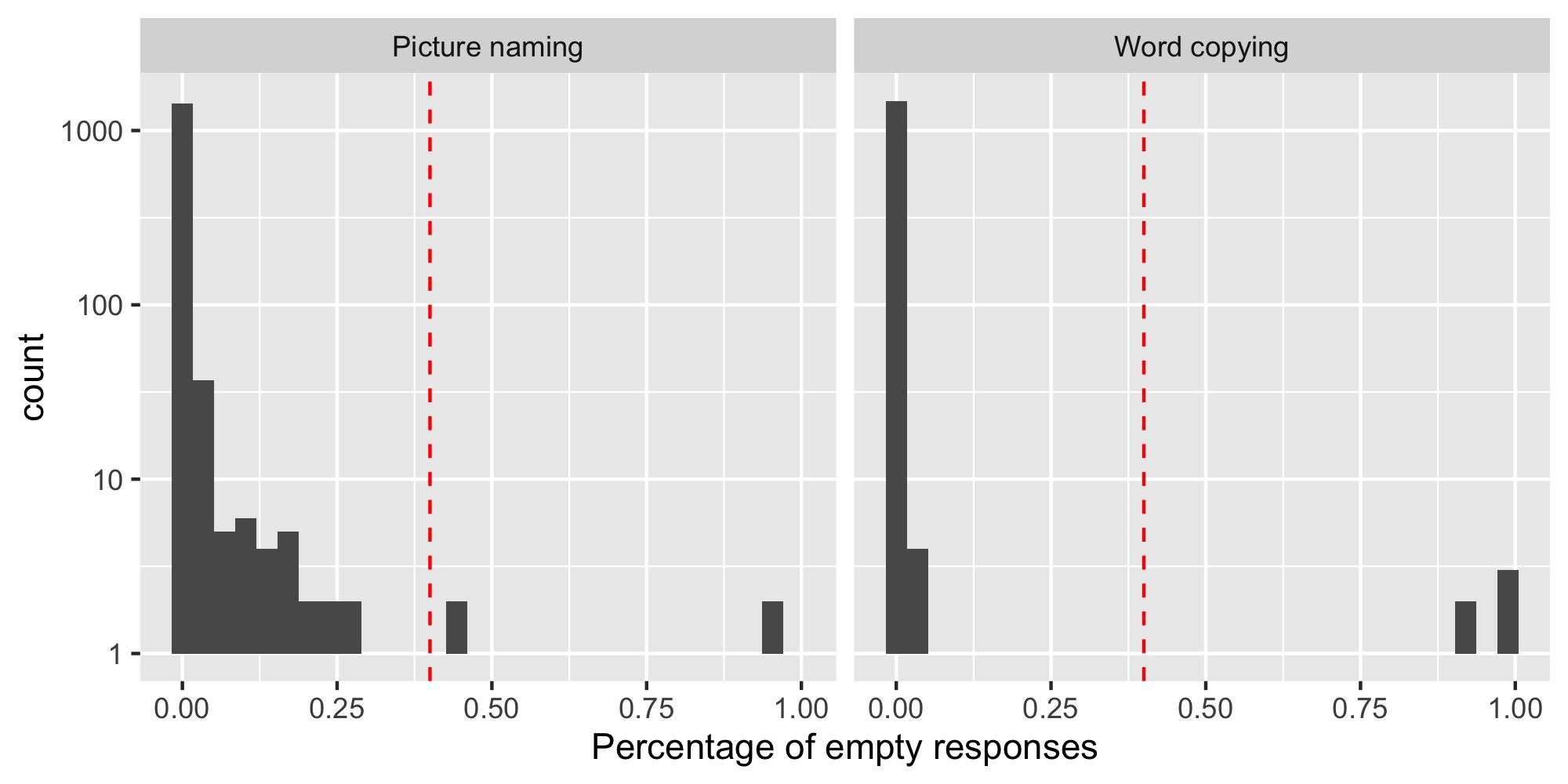

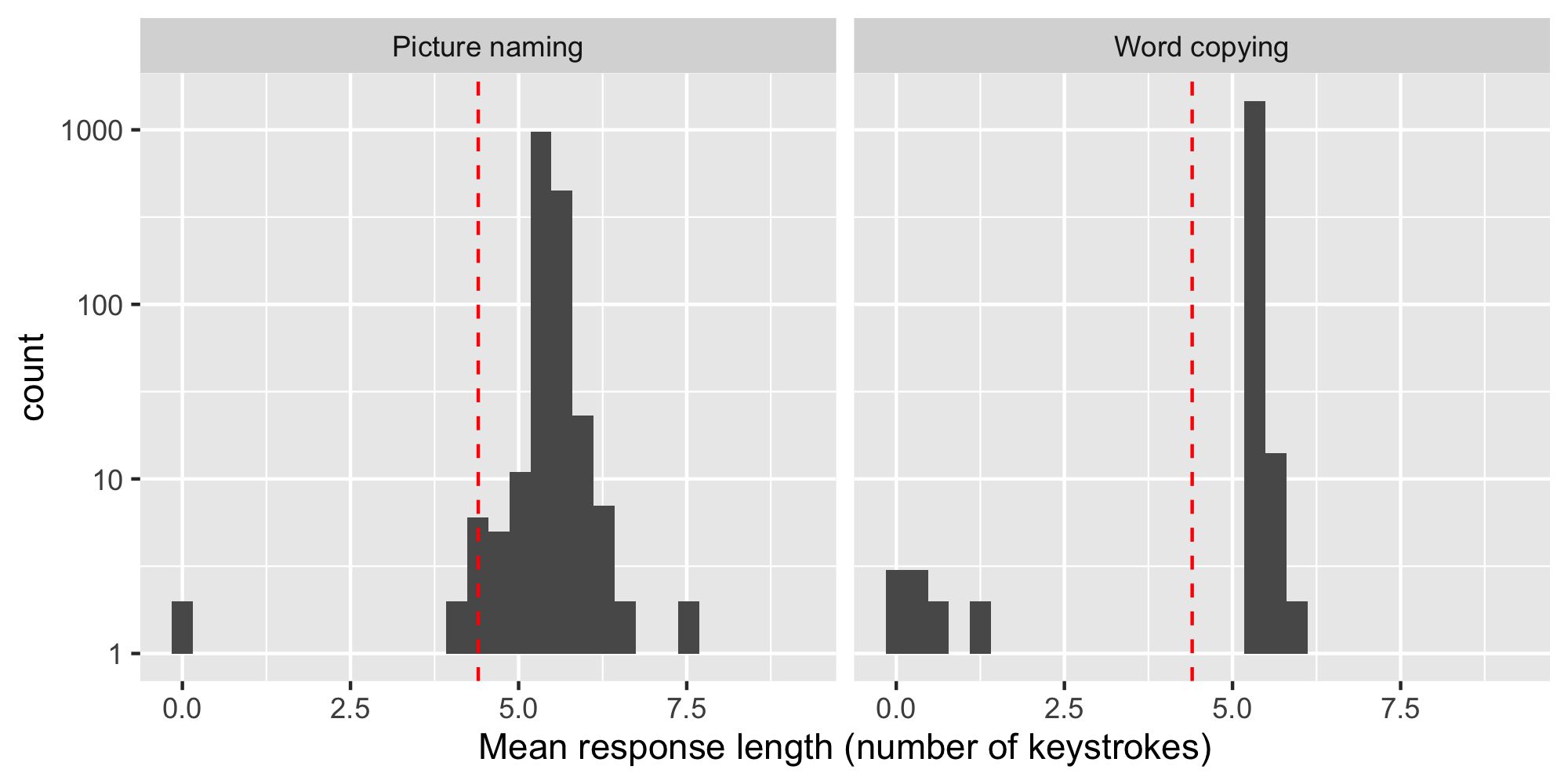

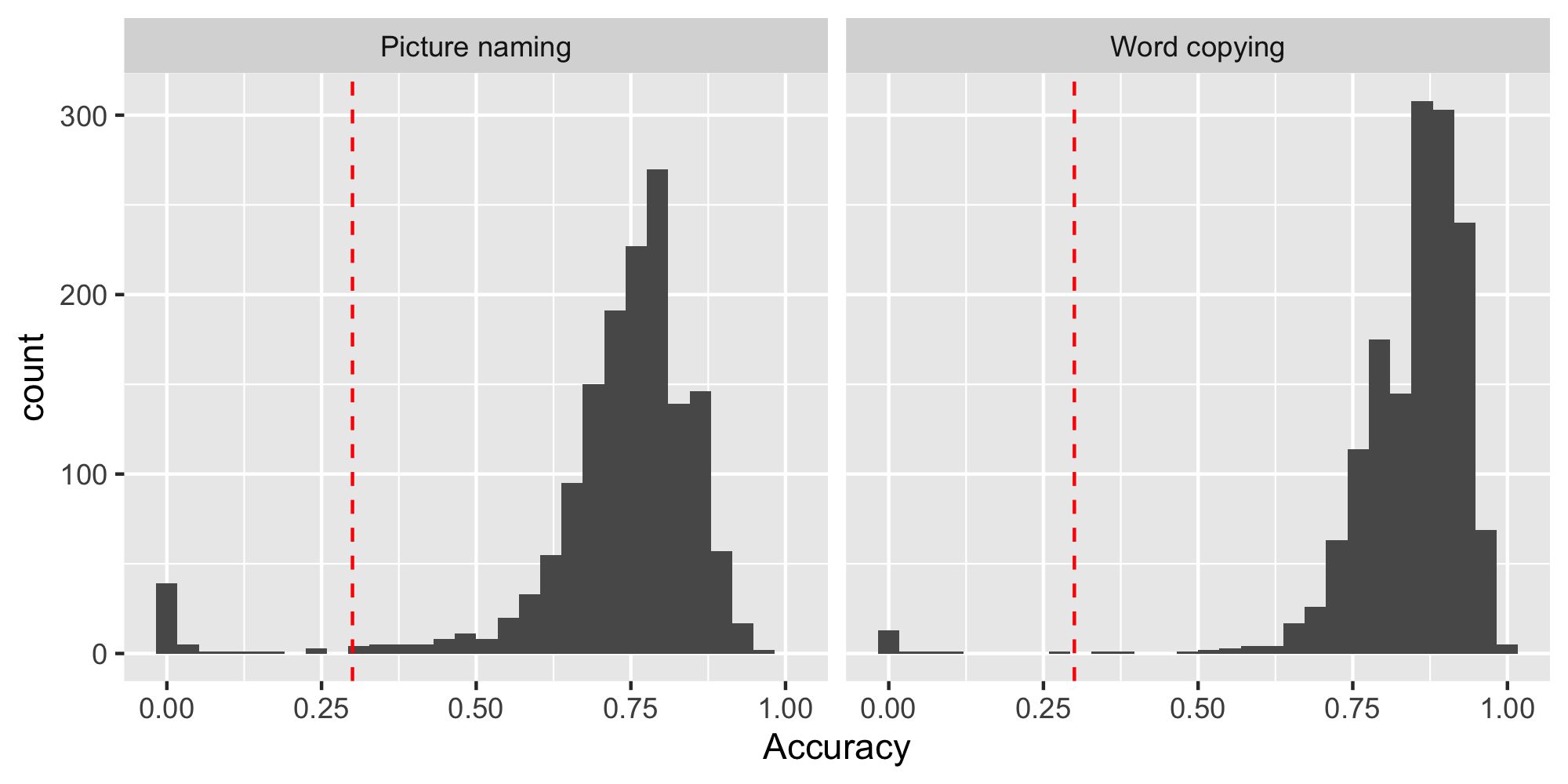

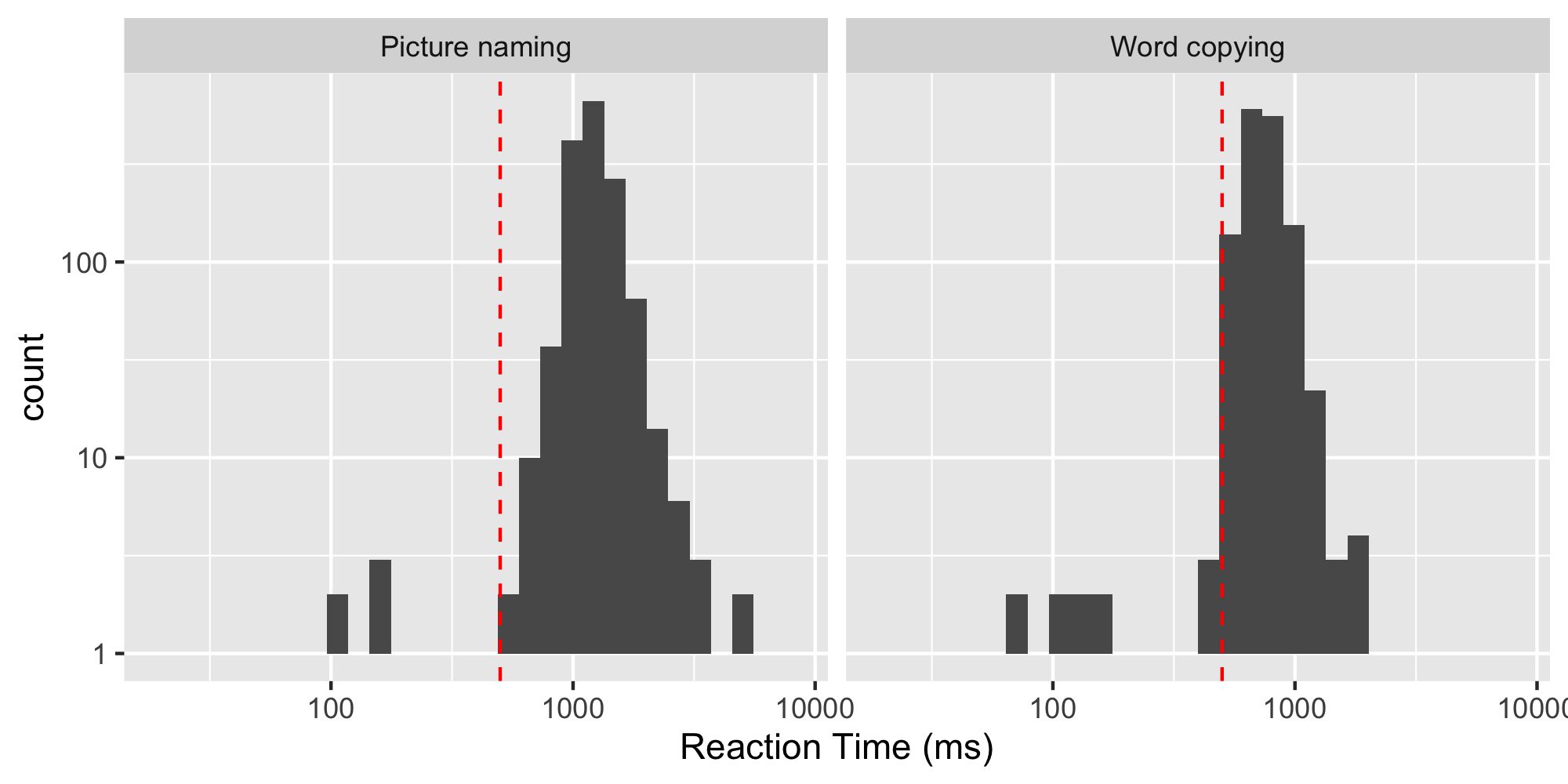


*Figure S2: Histograms of the percentage of empty responses, response length (in number of keystrokes), accuracy rates, and reaction times for the picture naming and word copying tasks. Red vertical line indicates the cut-off used for data rejection. Y-axis for graphs 1, 2, and 4, and x-axis for graph 4 is expressed in log-scale for visualization purposes.*

**Appendix 3: Time spent on each device**

The histogram of the daily time participants reported spending typing on a computer, on a mobile device, or handwriting is plotted in Figure S3.


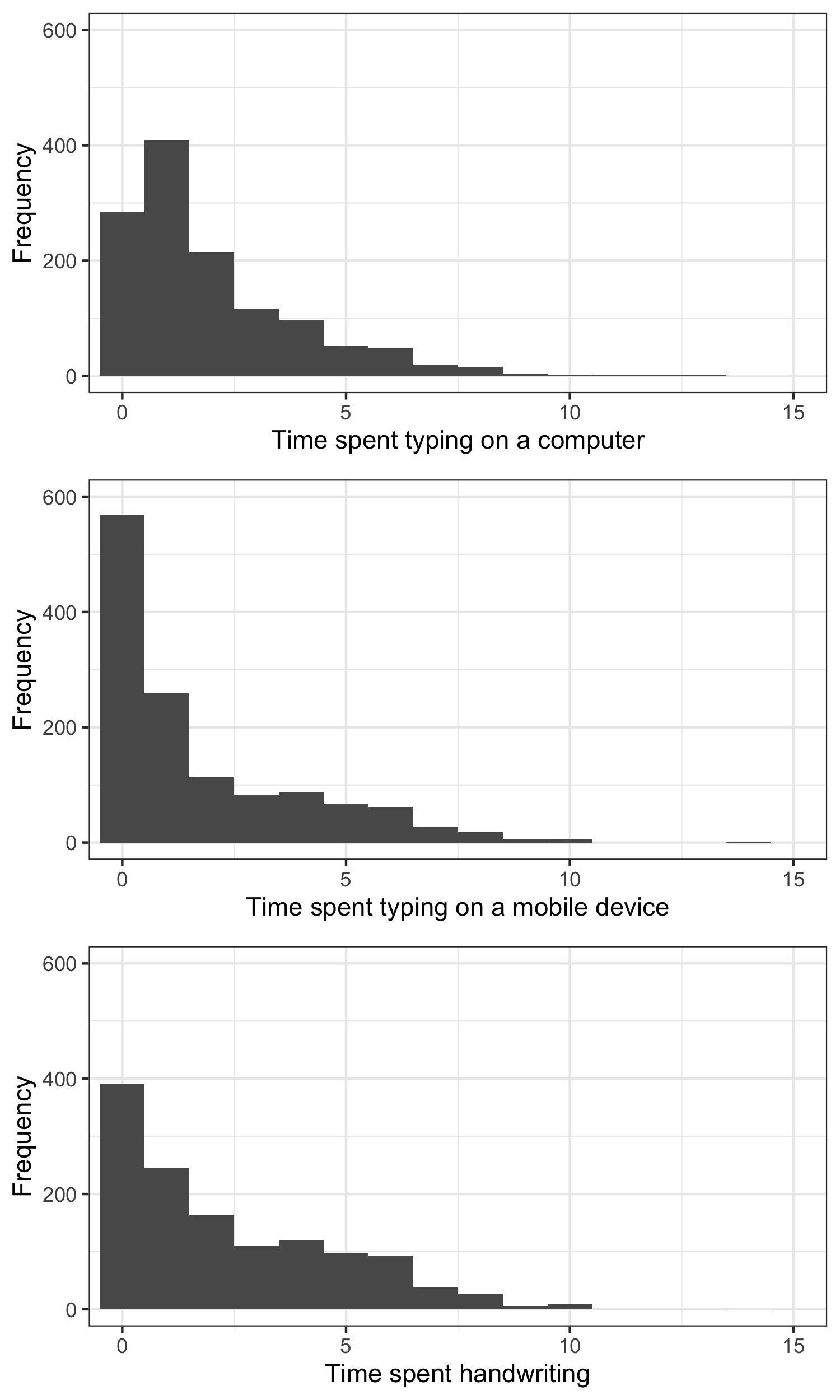


*Figure S3: Time spent handwriting and typing on each type of device in hours per day.*

**Appendix 4: Performance groups characteristics (RQ1)**

*Table S3: Descriptive statistics of typing speed and accuracy of the full sample and of each performance group selected.*

|  | Mean | Median | Q1-Q3 | Centile 5 -95 | Min-Max |
| --- | --- | --- | --- | --- | --- |
| Typing speed (wpm) | 65.0 | 63.2 | 52.5-76.3 | 38.7-97.2 | 22.4-137.9 |
| *High performers* | 79.9 | 88.5 | 68.8-88.5 | 56.9-106.8 | 42.9-137.9 |
| *Low performers* | 54.1 | 62.7 | 44.4-62.7 | 34.6-78.8 | 22.4-92.9 |
| Accuracy (%) | 84.5 | 85.5 | 81.1-88.9 | 73.1-92.4 | 45.7-96.1 |
| *High performers* | 88.1 | 88.6 | 85.8-90.1 | 81.7-93.6 | 76.0-96.1 |
| *Low performers* | 79.4 | 80.1 | 75.7-84.0 | 68.5-88.8 | 45.7-92.8 |

**Appendix 5: Results of typing habits analysis (RQ2)**

*Table S4: Results of regression analysis for daily time typing.*

|  | Estimate | Std Error | t | Pr(>\|t\|) | (sig.) |
| --- | --- | --- | --- | --- | --- |
| (Intercept) | 0.159 | 0.190 | 0.837 | 0.403 |  |
| Performance group | 0.385 | 0.071 | 5.442 | <.001 | *** |
| Age | -0.012 | 0.008 | -1.447 | 0.148 |  |
| Gender | 0.261 | 0.073 | 3.58 | <.001 | *** |

*Table S5: Results of regression analysis for years of practice.*

|  | Estimate | Std Error | t | Pr(>\|t\|) | (sig.) |
| --- | --- | --- | --- | --- | --- |
| (Intercept) | -1.558 | 0.694 | -2.245 | 0.025 | * |
| Performance group | 0.659 | 0.262 | 2.519 | 0.0119 | * |
| Age | 0.543 | 0.030 | 18.005 | <.001 | *** |
| Gender | -1.199 | 0.271 | -4.425 | <.001 | *** |

*Table S6: Results of regression analysis for number of fingers used for typing.*

|  | Estimate | Std Error | t | Pr(>\|t\|) | (sig.) |
| --- | --- | --- | --- | --- | --- |
| (Intercept) | 7.918 | 0.344 | 23.022 | <.001 | *** |
| Performance group | 0.401 | 0.130 | 3.093 | 0.0021 | ** |
| Age | -0.036 | 0.015 | -2.408 | 0.0163 | * |
| Gender | -0.383 | 0.134 | -2.859 | 0.0044 | ** |

*Table S7: Results of regression analysis for deliberate practice.*

|  | Estimate | Std Error | t | Pr(>\|t\|) | (sig.) |
| --- | --- | --- | --- | --- | --- |
| (Intercept) | -1.938 | 0.409 | -4.743 | <.001 | *** |
| Performance group | 0.055 | 0.144 | 0.385 | 0.700 |  |
| Age | 0.064 | 0.018 | 3.551 | <.001 | *** |
| Gender | 0.020 | 0.148 | 0.138 | 0.890 |  |

*Table S8: Results of regression analysis for lecture note-taking.*

|  | Estimate | Std Error | t | Pr(>\|t\|) | (sig.) |
| --- | --- | --- | --- | --- | --- |
| (Intercept) | 0.559 | 0.420 | 1.331 | 0.183 |  |
| Performance group | -0.934 | 0.145 | -6.439 | <.001 | *** |
| Age | 0.028 | 0.019 | 1.496 | 0.135 |  |
| Gender | -0.613 | 0.151 | -4.058 | <.001 | *** |

*Table S9: Results of regression analysis for looking at hands.*

|  | Estimate | Std Error | Z | Pr(>\|Z\|) |  |
| --- | --- | --- | --- | --- | --- |
| y>=rarely | 1.350 | 0.365 | 3.7 | <.001 | *** |
| y>=often | -1.237 | 0.358 | -3.46 | <.001 | *** |
| y>=always | -3.755 | 0.392 | -9.58 | <.001 | *** |
| Performance group | -1.309 | 0.139 | -9.44 | <.001 | *** |
| Age | 0.063 | 0.016 | 4.01 | <.001 | *** |
| Gender | 0.188 | 0.138 | 1.36 | 0.173 |  |

**Appendix 6: Analysis of accuracy rates (RQ3)**

Accuracy rates were fitted with the same predictors than RT but using a binomial model. Errors that included an attempt at correction (whether successful or not) were grouped with errors, and were contrasted to correct trials.

Results are presented in Table S10. Like for RTs and IKIs, accuracy rates revealed a main effect of performance group. Word Frequency, Length, Laterality, and Bigram Frequency all had significant main effects. Significant interactions with Performance group were observed for Length (in picture naming) and Word frequency (in word copying), such that high performers had a stronger effect of word frequency and a lower effect of length than low performers.

Some predictors had different effects according to the task. In particular, bigram frequency had a facilitatory main effect in word copying but an inhibitory effect in picture naming.

*Table S10: Results of mixed-effect model on accuracy rates in the picture naming and word copying tasks.*

|  | PICTURE NAMING | | | |  | WORD COPYING | | | |  |
| --- | --- | --- | --- | --- | --- | --- | --- | --- | --- | --- |
|  | ß | SE | z | p |  | ß | SE | z | p |  |
| (Intercept) | 1.1 | 0.018 | 60.159 | <.001 | *** | 1.56 | 0.021 | 74.89 | <.001 | *** |
| Performance group | 0.14 | 0.026 | 5.367 | <.001 | *** | 0.464 | 0.032 | 14.40 | <.001 | *** |
|  |  |  |  |  |  |  |  |  |  |  |
| Word Frequency (log) | 0.133 | 0.014 | 9.52 | <.001 | *** | 0.030 | 0.015 | 1.96 | 0.050 | * |
| Word Frequency x Performance group | 0.028 | 0.020 | 1.367 | 0.172 |  | 0.078 | 0.024 | 3.30 | <.001 | *** |
|  |  |  |  |  |  |  |  |  |  |  |
| Bigram Frequency (log) | -0.067 | 0.014 | -4.622 | <.001 | *** | 0.067 | 0.016 | 4.13 | <.001 | *** |
| Bigram Frequency x Performance group | -0.0085 | 0.021 | -0.408 | 0.684 |  | 0.013 | 0.025 | 0.52 | 0.604 |  |
|  |  |  |  |  |  |  |  |  |  |  |
| Length | -0.149 | 0.014 | -10.782 | <.001 | *** | -0.277 | 0.015 | -18.07 | <.001 | *** |
| Length x Performance group | 0.053 | 0.020 | 2.639 | 0.0083 | ** | 0.014 | 0.024 | 0.58 | 0.561 |  |
|  |  |  |  |  |  |  |  |  |  |  |
| Transition Percentage | -0.0043 | 0.014 | -0.312 | 0.755 |  | -0.042 | 0.015 | -2.70 | 0.0069 | ** |
| Transition Perc. x Performance group | 0.030 | 0.020 | 1.537 | 0.124 |  | 0.021 | 0.024 | 0.87 | 0.386 |  |
|  |  |  |  |  |  |  |  |  |  |  |
| Laterality | 0.084 | 0.027 | 3.098 | 0.00195 | ** | -0.093 | 0.030 | -3.09 | 0.0020 | ** |
| Laterality x Performance group | 0.065 | 0.039 | 1.655 | 0.098 | . | 0.034 | 0.046 | 0.73 | 0.463 |  |
|  |  |  |  |  |  |  |  |  |  |  |
| Trial | -0.0095 | 0.013 | -0.716 | 0.474 |  | -0.065 | 0.015 | -4.40 | <.001 | *** |
| Trial x Performance group | -0.0000071 | 0.019 | 0 | 1.00 |  | -0.018 | 0.023 | -0.78 | 0.437 |  |

**Appendix 7: Complementary analysis 1 – performance group by fifths**

We created performance groups based on a split of the initial distribution in fifths instead of thirds. We ran the same statistical models as in the main analyses but we considered only the first and last fifths of the distribution. This led to performance groups of 260 participants each.

The main effects observed for the analysis of typing habits and performance groups were maintained, as can be appreciated in the graphs below (Figure S4).


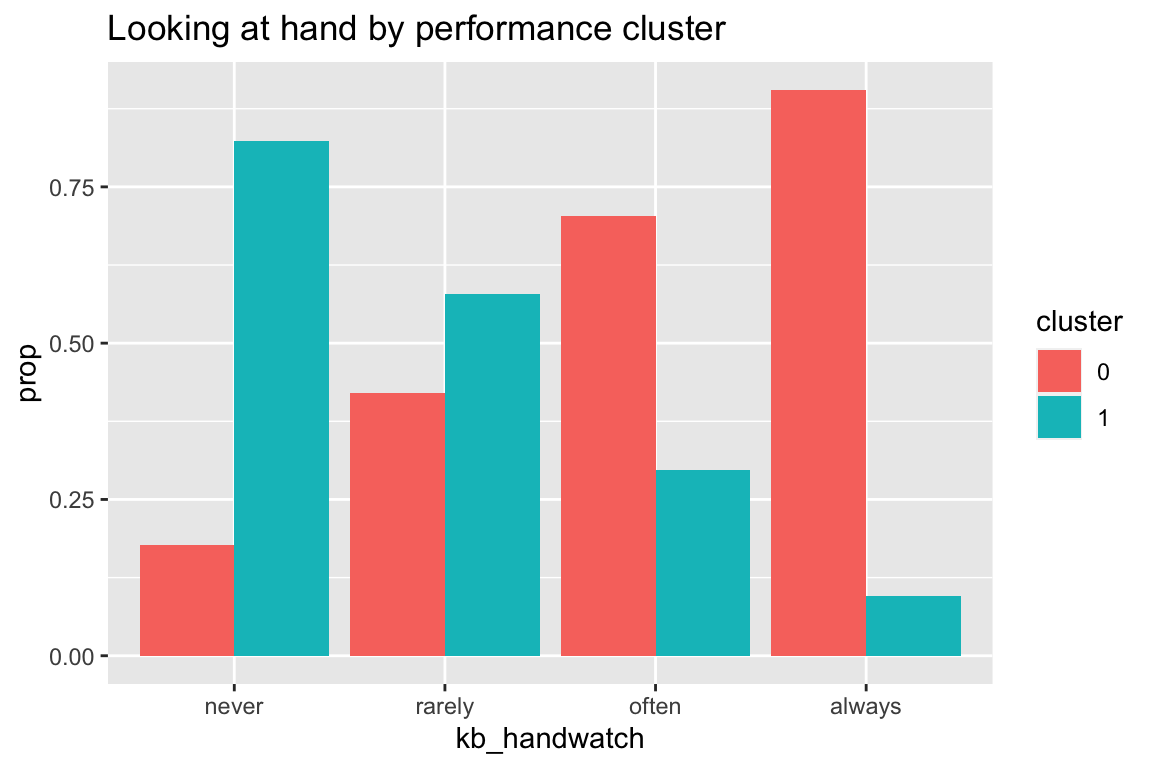

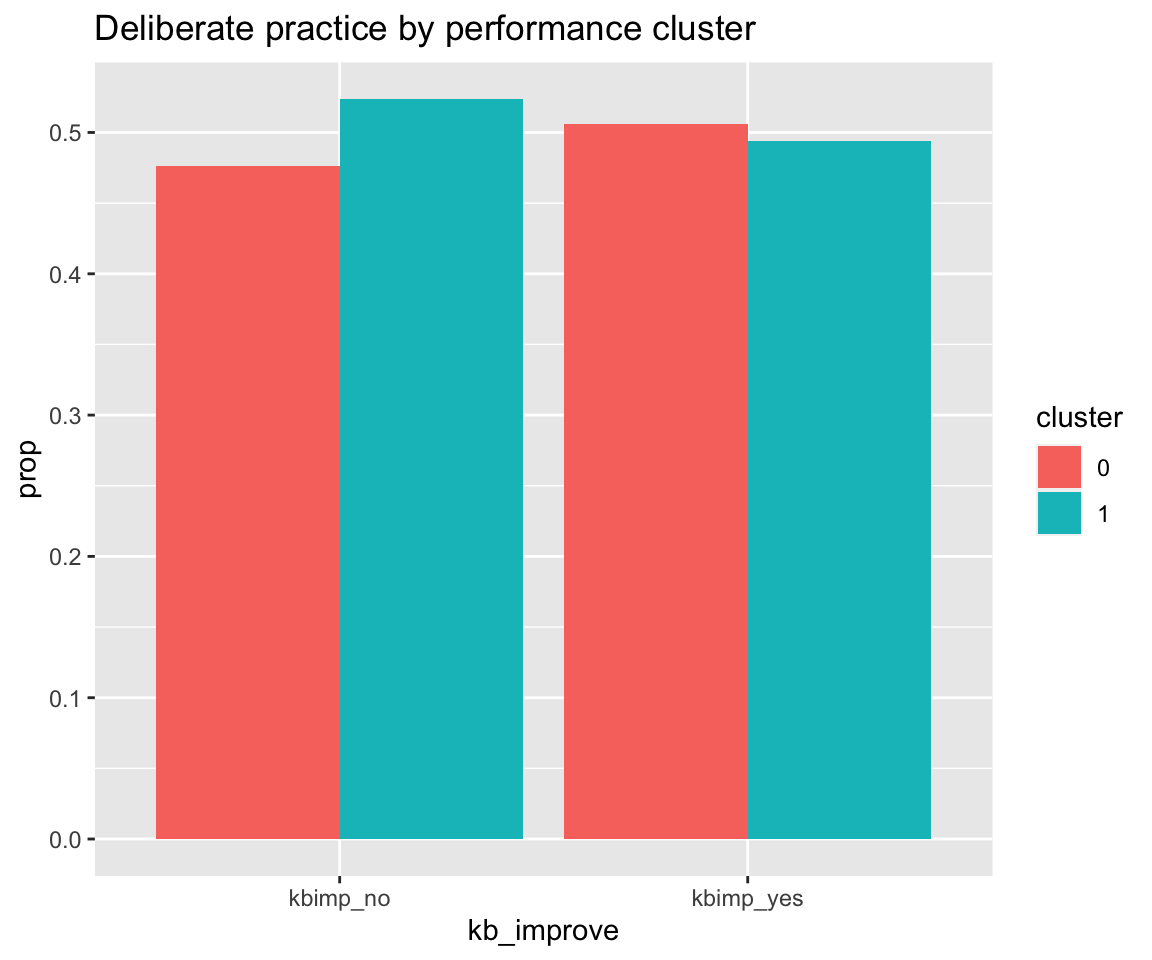


Figure S4: Left: Distribution of looks at hand in the two finger groups. Right: Distribution of deliberate practice in the two finger groups

The main effects of stimuli characteristics were also observed on such a subsample. In particular, on IKIs, effects were similar, in the same direction, in the same range, and were also significant (except for Trial on picture naming and Word Frequency for word copying), as can be seen in the table R2 below.

Table S11: Results of the mixed-model analysis on IKI for picture naming (left) and word copying (right) using finger groups.

|  | Word copying | | |  | Picture naming | | | |
| --- | --- | --- | --- | --- | --- | --- | --- | --- |
|  | ß | t | p |  | ß | t | p |  |
| (Intercept) | 4.980 | 374.78 | <.001 | *** | 4.990 | 380.31 | <.001 | *** |
| Word Frequency (log) | -0.038 | -4.99 | <.001 | *** | -0.036 | -4.90 | <.001 | *** |
| Length | 0.032 | 4.25 | <.001 | *** | 0.030 | 4.13 | <.001 | *** |
| Position | -0.042 | -25.09 | <.001 | *** | -0.043 | -24.23 | <.001 | *** |
| Transition Type | 0.164 | 47.25 | <.001 | *** | 0.169 | 46.30 | <.001 | *** |
| Bigram Frequency (log) | -0.054 | -30.27 | <.001 | *** | -0.057 | -30.32 | <.001 | *** |
| Trial | 0.007 | 4.25 | <.001 | *** | 0.003 | 1.66 | 0.097 | . |
| Performance group | -0.349 | -22.59 | <.001 | *** | -0.356 | -22.91 | <.001 | *** |
| Word Frequency x Performance group | 0.004 | 1.65 | 0.099 | . | -0.001 | -0.54 | 0.593 |  |
| Length x Performance group | -0.018 | -7.73 | <.001 | *** | -0.018 | -7.44 | <.001 | *** |
| Position x Performance group | -0.010 | -4.41 | <.001 | *** | -0.010 | -4.10 | <.001 | *** |
| Transition type x Performance group | 0.105 | 24.40 | <.001 | *** | 0.101 | 22.23 | <.001 | *** |
| Bigram Frequency x Performance group | 0.008 | 3.78 | <.001 | *** | 0.008 | 3.31 | <.001 | *** |
| Trial x Performance group | 0.002 | 1.04 | 0.300 |  | 0.000 | 0.17 | 0.865 |  |

**Appendix 8: Complementary analysis 2 – by finger use**

We divided the distribution of self-reported number of fingers used in two, with 7 as the cut-off to get groups of around 500 participants each (see Figure S5 below). Figure S5 (right panel) below shows the relationship between typing speed and accuracy (similar to Figure 3 of the manuscript) as a function of finger groups instead of performance groups. As can be seen from the distribution plot, the effect of typing style on speed, although present, isn’t massive. For instance, we observed a significant correlation between self-reported number of fingers and speed, r = 0.157, p <.001, consistent with Dhakal et al. (2018) where the correlation between typing speed and self-reported number of fingers was r = 0.38. It is important to note that their distribution of number of fingers used was somewhat different from ours. The highest proportion of their population (47%) reported using 9-10 fingers. In our case, the majority of our typists (40%) reported using between 6 and 8 fingers. This relative homogeneity in the number of fingers used could explain that typing style isn’t a stronger predictor of typing performance in our sample.


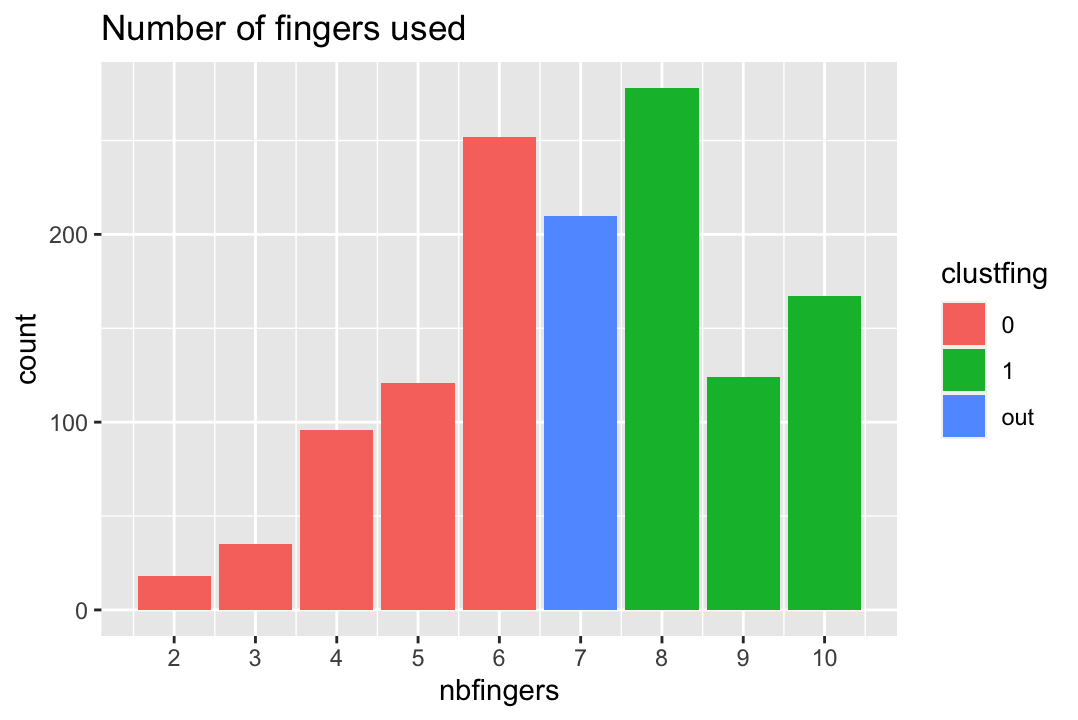

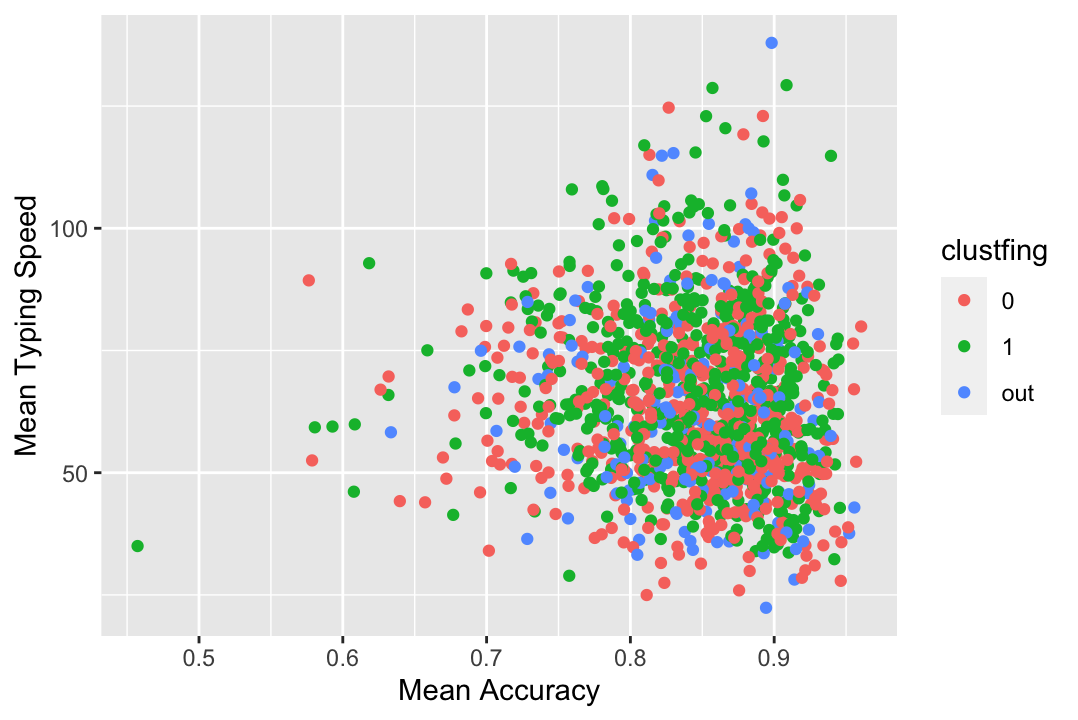


Figure S5: Left: Distribution of number of fingers used as self-reported by participants. Colors correspond to the grouping performed (0: 6 fingers or less, 1: 8 fingers or more). Right: Mean typing speed as a function of accuracy in the sentence copying task. Color coding corresponds to the finger groups defined according to the number of fingers used.

We ran exploratory analysis with these groups instead of the performance groups.

For the effects of typing practice and habits, the new analysis replicated the main effects we reported, as can be seen on the figures below (Figure S6) for looks at hand (significant effect of number of fingers used) and deliberate practice (non-significant effect of number of fingers used).


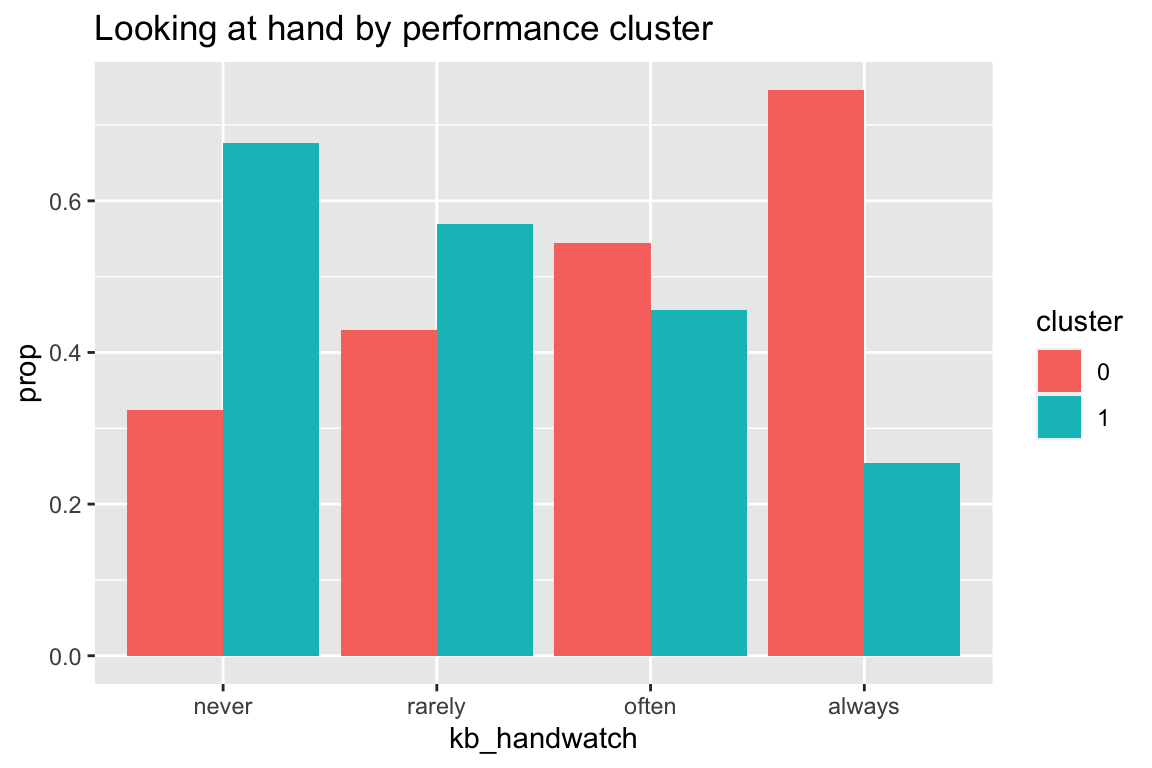

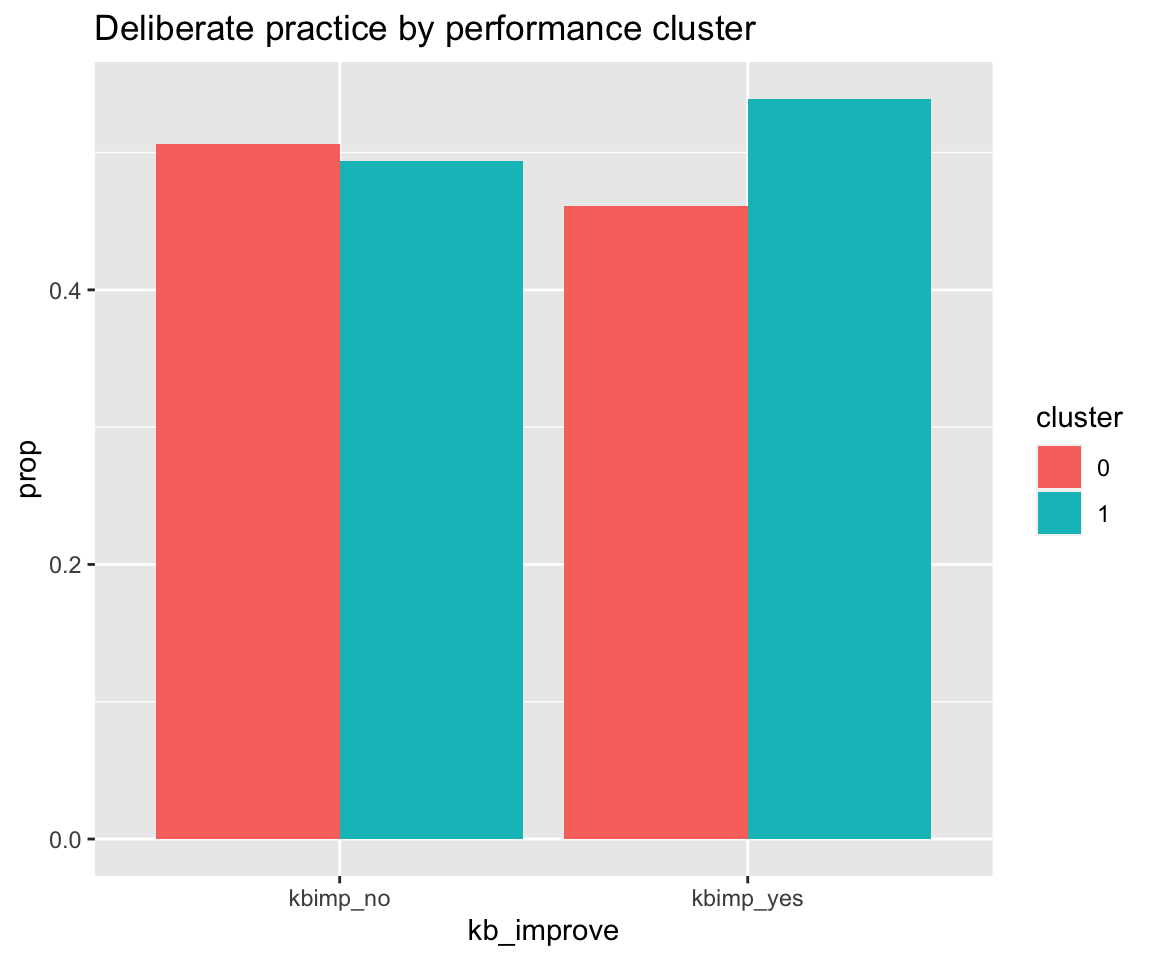


Figure S6: Left: Distribution of looks at hand in the two finger groups. Right: Distribution of deliberate practice in the two finger groups

For the effects of experimental manipulations in the picture naming and copy-typing tasks on IKI with finger groups, we found that the estimate of the main effect of cluster was significant for both tasks but smaller than with performance groups (estimates: -0.047 and -0.054 vs estimates -0.29 and -0.29 in the initial analysis). There were some differences in the interaction between finger groups and the effects of interests (see Table S12 below). In picture naming, there was no interaction of group with Length or Position anymore, and the interactions of group with Transition types and Bigram Frequency were in the other direction. In word copying, there was no interaction of group with Length anymore, the interaction of group with Position was stronger, and again the interactions of group with Transition types and Bigram Frequency were in the other direction. Similar results were obtained when using number of fingers as a continuous variable instead of making two groups.

The interactions observed in both tasks can be interpreted as such: typists that use more fingers show a stronger effect of bigram frequency, but a smaller effect of transition type than those using less fingers. They are plotted in Figure S7.

Table S12: Results of the mixed-model analysis on IKI for picture naming (left) and word copying (right) using finger groups.

|  | Picture naming | | | | Word copying | | | |
| --- | --- | --- | --- | --- | --- | --- | --- | --- |
|  | *ß* | *t* | *p* |  | *ß* | *t* | *p* |  |
| (Intercept) | 4.855 | 346.10 | <.001 | *** | 4.853 | 348.03 | <.001 | *** |
| Word Frequency (log) | -0.038 | -5.12 | <.001 | *** | -0.038 | -5.03 | <.001 | *** |
| Length | 0.021 | 2.88 | 0.0052 | ** | 0.024 | 3.20 | 0.0020 | ** |
| Position | -0.045 | -29.37 | <.001 | *** | -0.041 | -28.61 | <.001 | *** |
| Transition Type | 0.226 | 71.61 | <.001 | *** | 0.220 | 74.19 | <.001 | *** |
| Bigram Frequency (log) | -0.048 | -29.63 | <.001 | *** | -0.044 | -29.11 | <.001 | *** |
| Trial | 0.005 | 3.44 | <.001 | *** | 0.007 | 4.89 | <.001 | *** |
| Finger group | -0.047 | -2.86 | 0.0043 | ** | -0.054 | -3.36 | <.001 | *** |
| Word Frequency x Finger group | 0.001 | 0.65 | 0.516 |  | 0.002 | 1.23 | 0.217 |  |
| Length x Finger group | 0.000 | -0.10 | 0.919 |  | -0.001 | -0.71 | 0.479 |  |
| Position x Finger group | -0.004 | -1.77 | 0.076 | . | -0.008 | -4.21 | <.001 | *** |
| Transition type x Finger group | -0.018 | -4.74 | <.001 | *** | -0.010 | -2.88 | 0.0040 | ** |
| Bigram Frequency x Finger group | -0.010 | -4.94 | <.001 | *** | -0.012 | -6.36 | <.001 | *** |
| Trial x Finger group | -0.002 | -1.23 | 0.217 |  | 0.001 | 0.61 | 0.541 |  |


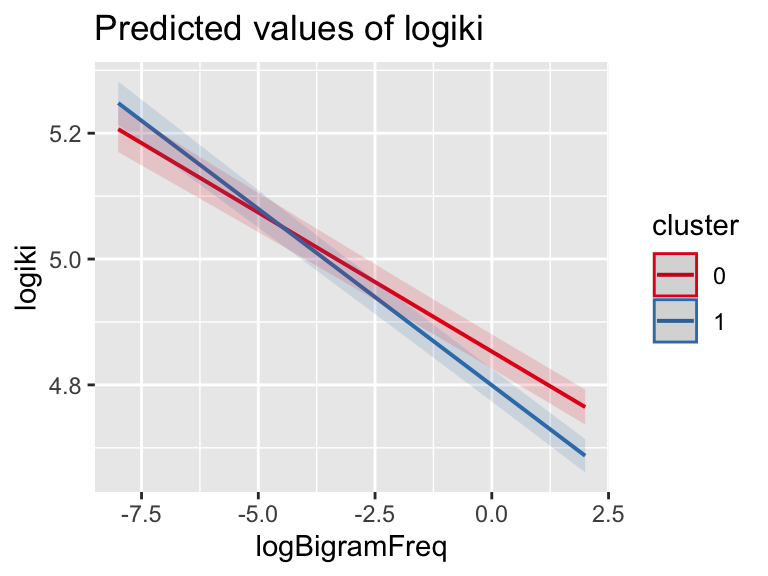

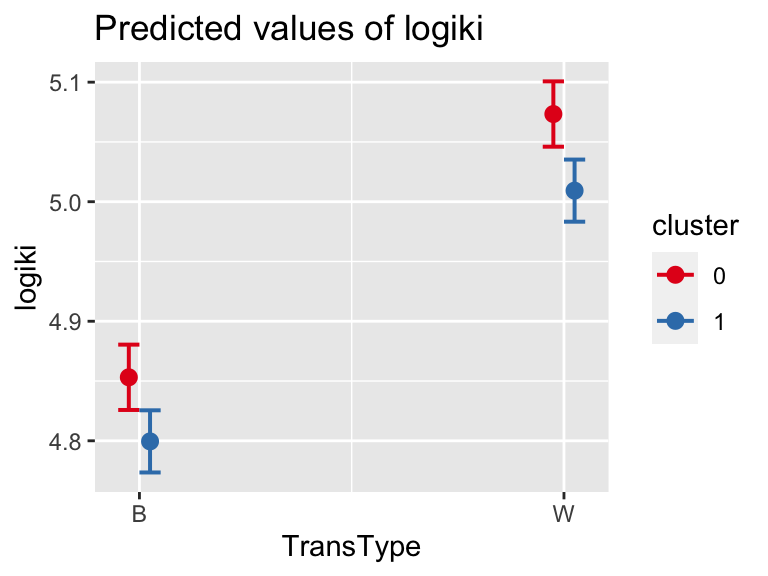


Figure S7: Left: interaction between finger group and bigram frequency. Right: interaction between finger group and transition type.
